# Hospital‐Based Cross‐Sectional Study of *Burkholderia pseudomallei* Seroreactivity Among Febrile Patients in Northernmost Vietnam: Near‐Neighbor Bloodstream Isolates, Environmental Correlates, and Spatial Clustering

**DOI:** 10.1029/2025GH001606

**Published:** 2026-05-30

**Authors:** Morgan C. Metrailer, Tran Thi Le Quyen, Khang Van Pham, Tan Minh Luong, Treenate Jiranantasak, Andrew P. Bluhm, Frank J. Tuozzo, Thi Thu Ha Hoang, Minh Hoa Luong, Bich Ngoc Do, Thanh Hai Pham, Madison E. A. Harman, Michael H. Norris, Trinh Thanh Trung, Jason K. Blackburn

**Affiliations:** ^1^ Spatial Epidemiology & Ecology Research Laboratory Department of Geography University of Florida Gainesville FL USA; ^2^ Emerging Pathogens Institute University of Florida Gainesville FL USA; ^3^ VNU ‐ Institute for Microbiology and Biotechnology Vietnam National University Hanoi Vietnam; ^4^ National Institute of Hygiene and Epidemiology Hanoi Vietnam; ^5^ Pathogen Analysis and Translational Health Group School of Life Sciences University of Hawaiʻi at Mānoa Honolulu HI USA

**Keywords:** melioidosis, vietnam, Burkholderia pseudomallei, disease prevalence, spatial analysis, infectious diseases

## Abstract

Melioidosis is a potentially fatal tropical disease caused by the environmentally mediated bacterium, *Burkholderia pseudomallei*. In Vietnam, melioidosis is not nationally reportable and little is known regarding its epidemiology and distribution in the northernmost mountainous provinces. To address this gap, a hospital‐based cross‐sectional study was conducted across six northernmost provinces of Vietnam: Son La, Dien Bien, Lai Chau, Lao Cai, Ha Giang, and Cao Bang. Objectives were to (a) elucidate the distribution and environmental correlates of *B. pseudomallei* seroreactivity, and (b) identify bacteria isolated from subset of admitted patients. *Burkholderia pseudomallei*‐specific IgG ELISAs were used to identify elevated serological responses in febrile (≥38°C, unknown cause) hospital patients (*n* = 2,440; 2020–2023). Exploratory blood culturing and whole genome sequencing (WGS) from a small subset of patients was performed. A commune‐scale presence‐absence analysis examined environmental associations to seropositivity via a generalized linear model with Moran's eigenvector spatial filtering. Spatial distributions of the proportion of patients IgG seropositive (+2 SD above mean) to Hcp1/OPS were smoothed using spatial Bayes and clusters were detected with local Moran's I. WGS revealed *B. pseudomallei* near‐neighbor species in some blood cultures; organisms that persist in similar environments, infect opportunistically, and are difficult to diagnose. A seasonal pattern was identified with more seropositivity among febrile hospital patients admitted in the wet season. At commune‐scale, seropositivity correlated to higher rainfall averages, proximity to major waterways, and low soil nutrients. Seropositivity was spatially heterogeneous and several spatial clusters (seropositivity hotspots) were detected, indicating a need for local or regional surveillance.

## Plain Language Summary

Melioidosis is an underreported disease that may cause severe and potentially fatal illness in humans. This disease is caused by an environmental bacterium that mainly transmits to humans and animals through contact with contaminated soil and water. Melioidosis is reported throughout much of Southeast Asia and Northern Australia; with recent studies targeting the burden and distribution in Vietnam. Little is known regarding this disease in the northernmost mountainous regions of Vietnam. Understanding seasonal and environmental patterns to the pathogen in this region can inform physicians and local communities on risk. From 2020–2023, we tested samples from hospital patients with unknown causes of disease. Exploratory genetic analysis found bacteria closely related to *Burkholderia pseudomallei* in some patients. Positive samples mostly occurred during the rainy season, showing a seasonal pattern. Patients from areas closer to major waterways with acidic, low‐nutrient soils were more likely to be positive. Positive patients were identified throughout the study area and several areas showed seropositivity hotspots based on spatial clustering analysis, suggesting the need for better monitoring outside cities.

## Key Points

- Potential *Burkholderia pseudomallei* detected in northernmost Vietnam; improved surveillance is suggested to detect true burden
- Patients living in communes with higher rainfall and low‐nutrient, acidic soils were more likely to show serological evidence of exposure
- Exploratory culturing found bacteria closely related to *B. pseudomallei* in a subset of patients

## 1 Introduction

Melioidosis is a potentially fatal infectious disease caused by the bacterium *Burkholderia pseudomallei* (Cheng & Currie, 2005; Whitmore & Krishnaswami, 1912). Melioidosis can be difficult to properly diagnose as signs and symptoms are highly variable, potentially affecting various organs or producing a systemic infection. Frequent misdiagnosis and manifestations similar to other illnesses has led to its moniker, the “great mimicker” (Yee et al., 1988). Classically, disease cases are associated with rice paddy field agriculture and flooding or monsoon events, where exposure to soil and water contaminants is increased (Cheng & Currie, 2005; Wiersinga et al., 2018). Severe cases of melioidosis have case fatality rates between 10%–50% in treated patients (Cheng & Currie, 2005), with greater mortality rates often associated with rurality and limited access to healthcare (White, 2003). Currently, no vaccine is available and antibiotic treatment can prove challenging as *B. pseudomallei* has innate and acquired antimicrobial resistance mechanisms–posing a risk for patients and a challenge for physicians (Schweizer, 2012; Wuthiekanun & Peacock, 2006).

*Burkholderia pseudomallei* is a motile, environmental saprophyte bacterium that is maintained in soil and water reservoirs. Human and animal infections with *B. pseudomallei* are transmitted through droplet or aerosol inhalation, ingestion, or through abrasions in the skin (Wiersinga et al., 2018). Though the role of animal *B. pseudomallei* infections in the maintenance and movement of the pathogen has been established (Norris et al., 2020), *B. pseudomallei* as a zoonosis is poorly understood, with limited information regarding transmission from animals or their products (Kwanhian et al., 2020; Sanchez‐Villamil et al., 2020). Disease prevalence is often greater in animals than in human populations because animals, such as swine or goats, are more likely to be exposed to environmental *B. pseudomallei* (Ekakoro et al., 2022; Norris et al., 2020). Humans are often considered accidental hosts of *B. pseudomallei*, with an environmental reservoir that can be replenished or redistributed by animal melioidosis (Sprague & Neubauer, 2004).

The *Burkholderia* genus contains human, animal, and plant pathogens that are geographically dispersed–typically occupying warm tropical regions (Duangurai et al., 2018; O’Rourke et al., 2017; Vandamme & Dawyndt, 2011). There are two species complexes within the genus: the *B. pseudomallei* complex (BPC) and the *B. cepacia* complex (BCC). The BPC is comprised of 8 species, including the virulent saprophytic *B. pseudomallei* and the obligate mammalian pathogen *B*. *mallei*. The BCC complex is comprised of 24 distinct species that occupy soil and water environments and often act as opportunistic pathogens (Eberl & Vandamme, 2016). Similarly, species within the genus *Pandoraea* co‐inhabit these environments and are noted multi‐drug resistant opportunistic pathogens that can be misidentified as *Burkholderia* spp. (Costello et al., 2011).

Species within the *Burkholderia* genus can be human public health concerns due to their intrinsic and acquired antibiotic resistance mechanisms; with a history of severe infections and increased risk in cystic fibrosis (CF) patients, diabetics, and immunocompromised individuals (B. J. Currie et al., 2004; Limmathurotsakul et al., 2010; Vandamme et al., 1997; Vandamme & Dawyndt, 2011). Many of the species in both complexes are soil‐dwelling saprophytes (e.g., *B. pseudomallei*, *B. thailandensis, B. cenocepacia*); *B. mallei* is a frank pathogen that cannot survive in the environment and has become host dependent due to genome reduction (Nierman et al., 2004; Vandamme & Dawyndt, 2011). The diverse genomic and phenotypic nature of *B. pseudomallei* allows it to adapt to nutrient limited and potentially adverse environments (both natural and man‐made) and various host intracellular systems (Duangurai et al., 2018; Galyov et al., 2010; O’Rourke et al., 2017). These characteristics are shared amongst other species in both complexes–however, the virulence, environmental prevalence, and aerosol transmission risk of *B. pseudomallei* has led to its classification as a potential bioterrorism agent and a Tier 1 select agent by the United States Centers for Disease Control and Prevention (CDC, 2009; Gilad et al., 2007).

The geographic distribution of melioidosis is widespread in certain environmental conditions, and likely expanding (Birnie et al., 2022; Limmathurotsakul et al., 2016). Melioidosis is “hyperendemic” to Northern Australia (Smith et al., 2018) and Thailand (Hinjoy et al., 2018; Kaewrakmuk et al., 2023), with many of the historical and current cases being reported in these regions. The disease is considered endemic to approximately 45 countries, spanning South America, parts of Africa (Birnie et al., 2022), and a majority of Southern and Southeast Asia (Limmathurotsakul et al., 2016). Systemic underreporting continues to obscure disease burden in non‐endemic and endemic countries, including Vietnam (Limmathurotsakul et al., 2016). In Vietnam, a high hospital burden of melioidosis was detected in its central region by the Research Network on Melioidosis and *B. pseudomallei* (RENOMAB)–a collaborative effort between some provincial hospitals and Vietnam National University (Norris et al., 2026; Trinh, Nguyen, et al., 2018). Despite its known endemicity across Southeast Asia, the disease history in Central Vietnam, and recent high profile child mortalities (Tran, Phan, et al., 2022), melioidosis is not a nationally reportable disease in the country.

In Vietnam, the efforts of RENOMAB have worked to elucidate the burden of disease by targeting acute infections of suspected melioidosis through clinical guided identification (Trinh, Nguyen, et al., 2018). While culture‐based detection is ideal for detecting acute infections, this method of detection has low sensitivity and can fail to detect infections in the absence of bacteremia (Limmathurotsakul et al., 2013; Suttisak et al., 2026). Furthermore, this method of diagnostics can prove costly, both in trained personnel and laboratory capacity. Thus, the use of serological methodologies, such as enzyme‐linked immunosorbent assays (ELISA) and other immune response diagnostics can work to elucidate the potential underlying burden and guide targeted surveillance efforts, particularly in resource‐limited settings (Haselbeck et al., 2022).

The epidemiology and geographic distribution of melioidosis in the northernmost provinces of Vietnam is poorly characterized (Limmathurotsakul et al., 2016). Understanding the distribution of seroreactivity and the associated environmental conditions in this region would benefit local and regional knowledge campaigns and clinical awareness, particularly for rural communities and occupationally exposed groups such as agricultural workers. Currently, the epidemiology of human melioidosis in Vietnam is guided by clinicians and the detection of acute infections. In the current work, the efforts are opportunistic and exploratory, aimed to complement current epidemiological work and guide future surveillance efforts in an under studied region. The objectives of this study were first to elucidate the distribution and environmental correlates of *B. pseudomallei* seroreactivity in the northernmost provinces of Vietnam; and second, to identify bacteria found in a subset of febrile hospital patients.

## 2 Materials and Methods

### 2.1 Ethics Statement

The parent study for sera collection underwent ethical review by the Institutional Review Board in Biomedical Research of the National Institute of Hygiene and Epidemiology (NIHE), Vietnam (IRB‐VN01057/IORG 0008555, Project IRB certificate number NIHE IRB‐03/2020) and the University of Florida, United States of America (USA) (UF IRB202003189). The current study for *B. pseudomallei* underwent ethical review by the University of Medicine and Pharmacy, Vietnam National University (IRB# IRB00013221) and the University of Florida, USA (IRB# IRB202301112). The transfer of serum aliquots derived from the parent study for use in the current study was explicitly reviewed and approved by the participating institutes.

### 2.2 Sample Collection and Management

**Figure 1:**
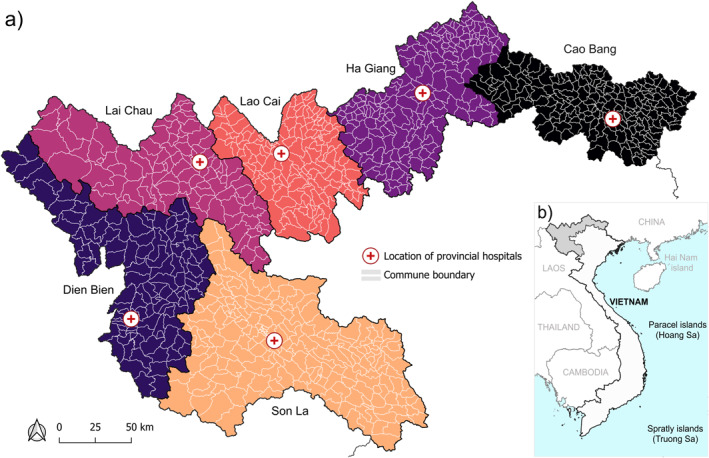
Vietnam provinces in the study area; Son La, Dien Bien, Lai Chau, Lao Cai, Ha Giang, and Cao Bang (a). Within province, commune boundaries (administrative level 3) are outlined in white. Provincial hospitals participating in this study are indicated (red cross symbol) (a). Inset map of mainland Vietnam with island and sea territories (b). The study area is shown in gray and bordering countries are identified (b). The Vietnamese administrative levels were in place before 1 July 2025.

This study follows the Strengthening the Reporting of Observational Studies in Epidemiology (STROBE) guidelines as defined for cross‐sectional studies; a checklist is provided in Table S1 (Elm et al., 2007). The study region included the six northernmost border provinces in Vietnam: Son La, Dien Bien, Lai Chau, Lao Cai, Ha Giang, and Cao Bang (Figure 1). As part of a large surveillance effort for exposure to other zoonoses, including *Bacillus anthracis* (anthrax) and *Brucella* spp (brucellosis), we conducted a hospital based cross‐sectional study using human serum and whole blood collected at six provincial hospitals between 2020 and 2023 from patients with undifferentiated febrile illnesses at time of collection (*n* = 2,956). Patients with a temperature over 38.0°C were considered febrile. This was convenience sampling with no exclusions based on age or clinical setting (inpatient, outpatient, and/or emergency setting); we cannot exclude the possibility that underrepresentation of groups with limited access and proximity to the major hospitals occurred. Repeat sampling of individuals was not performed and available patient‐level demographics were limited to age, geographic location, and gender; clinical data and information on health status, aside from fever, was not available. Of 2,956 enrolled patients, 2,440 (82.5%) had specimens (blood serum) available and were tested for *B. pseudomallei* seroreactivity; 392 had no specimens available, 103 had no geographic information, and 21 had no date of birth. Sample size was determined by the availability of specimens collected under the parent surveillance study; no a priori power calculation was performed for this study.

In accordance with sample storage guidelines for *Brucella* spp., serum and blood were collected in EDTA [ethylenediaminetetraacetic acid] and stored long‐term at −80°C (Navarro et al., 2004; Vrioni et al., 2008). Following immediate collection at district hospitals, specimens were stored at 4°C, while those at the general provincial hospital were stored at −20°C. Samples were transported on dry ice to Vietnam Centers for Disease Control (CDCs) and the National Institute of Hygiene and Epidemiology (NIHE) in Hanoi, Vietnam and stored at −80°C long term. For the current study, aliquots were transferred on dry ice to the Institute of Microbiology and Biotechnology (IMBT) at Vietnam National University in Hanoi and stored at −80°C prior to serological screening and bacterial culture. In total, serum samples underwent <3 freeze‐thaw cycles and were thawed overnight at 4°C prior to testing.

### 2.3 Serology

Human sera samples were tested for the presence of antibodies against *B. pseudomallei* antigens, type A outer polysaccharide (OPS) (a component of the lipopolysaccharide LPS) and the recombinant hemolysin coregulated protein 1 (Hcp1) using an enzyme‐linked immunosorbent assay (ELISA). Recombinant Hcp1 is the immunogenic component of the virulence cluster 1 type VI secretion system (T6SS) (Burtnick et al., 2011). Antigen purification was performed at the University of Florida's Emerging Pathogens Institute. The isolation of OPS from Bp82 Δ*wcb* mutant was performed following methods described in Norris et al. (2017), originally adapted from Lam et al. (2014). The Hcp1 antigen was prepared following the methods described in Norris et al. (2020). The plates were coated with 2 μg/mL OPS and 4 μg/mL purified Hcp1 overnight at room temperature before being washed three times with phosphate‐buffered saline (PBS). Plates were dried, sealed with parafilm, and stored at −80°C until shipment. Plates were shipped on ice using frozen gel packs to Hanoi, Vietnam and then stored at 4°C until used.

Serum from febrile patients was diluted 1:500 in blocking solution, 1X PBST (PBS with 0.005% Tween 20; Pierce 20X PBS Tween‐20 Buffer; Thermo Scientific) with 5% (*w/v*) of BLOTTO blocking grade non‐fat skim milk powder (Santa Cruz Biotechnology, Dallas, TX, USA). Blocking of ELISA plates was performed with the same solution for 1 hr before being inoculated with 200 μl of diluted serum for 1 hr at room temperature (RT). Then, plates were washed three times with blocking solution and 100 μl of 1:15,000 rabbit anti‐human IgG (H + L) secondary antibody, HRP (Thermo Fisher Scientific, Waltham, Massachusetts, USA) was incubated on the plates for 1 hr. Plates were washed three times with 1X PBST, then 100 μl of TMB substrate was added (1‐Step Ultra TMB ELISA, Thermo Fisher Scientific, Waltham, Massachusetts, USA) and allowed to develop for 30 min. Stop solution (1N HCl) was added (100 μl) and the resulting absorbance values were read using at *A*_450_ within 30 min. Both assays were run in duplicate, and the average value was calculated for each sample and assay.

### 2.4 Exploratory Blood Culturing, Polymerase Chain Reaction, and Phylogenetics

Following the first stage of serological testing (*n* < 300), blood samples from a small subset of seroreactive patients were blood cultured; 14 blood culture bottles were available for preliminary culturing efforts. As the blood in this study was collected as part of a targeted effort to identify *Brucella* species, they were collected in anticoagulant EDTA vacuum tubes and stored at −80°C. Here, an exploratory effort to isolate suspected *B*. *pseudomallei* was performed, despite limitations in available blood quantity and known challenges in isolating *Burkholderia* spp. from low volume blood isolations. Fourteen blood samples were selected from initial ELISA plates and blood bottles (BACT/ALERT FA Plus, Biomerieux, Marcy‐l'Étoile, France) were injected with approximately 1 ml of patient whole blood and incubated at 37°C for up to 96 h (Trinh, Hoang, et al., 2018). Bottles showing hemolysis and bacterial growth (*n* = 4) were streaked on to Ashdown's selective agar (Ashdown, 1979) and incubated at 37°C for up to 72 hr. Resulting bacterial colonies were Gram‐stained and compared to Gram staining imagery of *B. pseudomallei* Bp82 strain performed at University of Florida's Emerging Pathogens Institute. Genomic deoxyribonucleic acid (gDNA) was extracted from colonies with Gram‐negative rod‐shaped bacteria typical of *B. pseudomallei* using the Wizard® Genomic DNA Purification Kit (A1120, Promega, Madison, WI, USA). Isolates (*n* = 4) were tested for the presence of the *B. pseudomallei* TTS1 *orf2* using the established quantitative polymerase chain reaction (qPCR) assay (Novak et al., 2006). Blood bottles that showed no bacterial growth after 96 hr were not tested using the established qPCR assay.

To identify the species grown from the blood bottles on selective Ashdown's media, whole genome sequencing was performed on the extracted DNA isolates (*n* = 4) and downstream phylogenetic analyses were conducted. Library preparation and sequencing were performed following methods previously established by this group in the region (Metrailer et al., 2023; Norris et al., 2026). Briefly, libraries were prepared using the Nextera XT DNA Library Preparation Kit and Nextera XT Index Kit v2 (Illumina, San Diego, CA, USA). Quality and quantity of libraries was confirmed using a 2% (w/v) agarose gel stained with ethidium bromide and the QubitTM ds DNA High Sensitivity Assay Kit (Life Technologies Corporation, Eugene, OR, USA), respectively. Sequencing was performed on the Illumina MiSeq platform using the Miseq® v2 300 cycle PE kit with a 5% PhiX spike‐in (Illumina, San Diego, CA, USA). Raw read statistics were assessed with SeqKit (Shen et al., 2016).

Genomic assemblies were produced using a snakemake pipeline on the University of Florida's high performance supercomputer (HiPerGator). Within the pipeline, paired end reads files (FASTQ) were trimmed using trimmomatic [45] and a de novo genome assembly was produced using SPAdes with optimizer Unicycler [46,47]. Species identification was determined using the online PubMLST species identifier tool (Jolley et al., 2018). Reference quality genomes from the identified species, *Burkholderia gladioi*, and all species within the BPC and the BCC were retrieved from the National Center for Biotechnology Information (NCBI). A list of genomes used in the study are provided in Table S2. Sequence data sourced from this study have been deposited in the NCBI database under BioProject PRJNA1413423.

A maximum likelihood tree was produced using the Phylogenetic and Molecular Evolution (PhaME) (Shakya et al., 2020) analysis tool. As implemented within PhaME, core genome SNPs were identified using the alignment tool nucmer against the reference genome *B. pseudomallei* 1026b (Accession: SAMN03097397). The use of core genome SNPs naturally filters out larger recombinant or accessory genomic regions, and the resulting concatenated SNP alignment was used to infer a maximum‐likelihood phylogeny with RAxML, with statistical support via nonparametric bootstrapping (x100). The tree was rooted using *Pandoraea capi* strains as outgroup taxa, as this species falls outside the *Burkholderia* genus. All *Burkholderia* species, including *B. gladioli*, were treated as in‐group taxa. Tree visualization and figure production was performed with the Interactive Tree of Life (iTOL) (Letunic & Bork, 2024) and Inkscape v.1.4.2.

Following taxonomic identification of these isolates, whole cell lysate assays were prepared from the subset of isolates identified within the target genus (*Burkholderia*) as a limited examination of specificity of selected antigens in our ELISA and to assess potential cross reactivity. Following methods described in Tran, Nguyen, et al. (2022); fresh bacterial cells from isolated colonies were lysed, suspended in PBS, and coated overnight on 96‐well plates using the conditions described previously for the OPS and Hcp1 assays. In parallel, sera were repeated on the Hcp1 assay. Assays were run in duplicate including serum from the corresponding isolate‐source patients, a random selection of samples from the population (*n* = 92), and confirmed melioidosis‐positive (active infection) and melioidosis‐negative control sera. The average absorbance value was calculated for each sample and assay.

### 2.5 Seropositivity

Seropositivity was defined using distribution‐based thresholds derived from OPS and Hcp1 ELISA absorbance values, in the absence of assay‐specific positive and negative control sera. An assessment of the distribution of absorbance values overall and across different demographic strata found similar unimodal reactivity dynamics were present between groups (Figures S1–S6 in Supporting Information S1). This distribution‐based method of determining cutoff values for seropositive samples assumes that the majority of the population is seronegative, such that elevated absorbance values represent a minority of individuals with specific antibody reactivity to *B*. *pseudomallei* OPS or Hcp1 antigens (Weppelmann et al., 2018). While this methodology has limitations in the absence of local negative controls, this opportunistic exploratory study follows precedent from earlier work that utilizing these prototype assays in Vietnam (Norris et al., 2020). Liberal and conservative absorbance thresholds were set at two and three standard deviations (SD, σ) above the population mean (*µ*) to theoretically exclude ≥95% and ≥99.7% of seronegative individuals from being classified as seropositive, respectively (Ekakoro et al., 2022; Jacobson, 1998; Weppelmann et al., 2018). Because this is a febrile population, non‐specific immune activation and/or cross‐reactive antibodies could elevate background absorbance values, shifting μ and σ upward and potentially biasing seropositivity estimates (typically toward underestimation). To further address this potential bias, we defined seropositivity using two thresholds: individuals were classified as positive under the liberal cutoff if they exceeded the lower cutoff (absorbance ≥*µ* + 2σ) in either assay, or under the conservative cutoff if they exceeded the higher cutoff (absorbance ≥*µ* + 3σ) in either assay.

Characteristics of seropositive and seronegative individuals, for both liberal and conservative cutoffs, were compared using chi‐square tests (with continuity correction) for categorical variables. Individuals were stratified by gender, age group, and home province. Individuals missing gender information (*n* = 14) were not included in this comparative analysis. Age groups were produced based on known activity and behavioral patterns within groups, particularly for younger and older populations. Statistical significance was defined at *p* < 0.05. All analyses were conducted in RStudio version 2025.09.2 using the *tableone* package. The analyses were repeated for individual assays, OPS and Hcp1, with seropositive both cutoffs.

Rainfall and maximum temperature data were used to test for seasonality and *B. pseudomallei* seroreactivity in febrile patients. This analysis was exploratory because IgG seroreactivity does not establish the timing of exposure or true infection. Graphs of the monthly average rainfall and maximum temperature per province over the study period (2020–2022) were produced in RStudio version 2025.09.2. These values were plotted as means across all six provinces, with standard deviations representing the variability between provinces. Monthly seropositivity rates were calculated as the number of seropositive patients (using both cutoffs) divided by the total number of tested febrile patients and plotted as lines with a corresponding secondary (right) *y*‐axis.

### 2.6 Commune‐Level Environmental and Geographic Correlates

Spatial analyses in this study were performed at the third administrative level (commune) according to the Vietnamese administrative system in place before 1 July 2025, using administrative boundaries for the six provinces downloaded from gadm (https://gadm.org/) (Figure 1). All spatial analyses were performed using the EPSG:3405–VN‐2000/UTM zone 48N projection in QGIS version 3.34.1 Prizren. Patients were geocoded to the centroid of their home commune using QGIS. For the purposes of exploratory environmental and geographic analyses, presence communes were defined as having at least one patient that was IgG seropositive following either the liberal or conservative cutoff. Communes with no tested patients were excluded from analyses.

Environmental and geographic characteristics of the study area were derived from open web sources, reprojected, and aggregated to the commune level. These covariates were prespecified to assess potential geographic and ecological associations to elevated antibody responses to *B*. *pseudomallei*. Detailed descriptions of the data sources, spatial resolution, and temporal range are listed in Table S4 in Supporting Information S1. The monthly accumulated precipitation (mm) and soil moisture (mm) from 2020 to 2022 were summed and the values for each year were then aggregated (mean statistic) to the commune level for the entire study region using the zonal statistics routine in QGIS. The yearly average over the study period was determined. Additional soil parameters were investigated in this study: nitrogen content (cg/kg), organic carbon content (dg/kg), and soil pH, and the majority values were calculated for each commune (zonal statistics). Soil rasters were retrieved from SoilGrids (https://soilgrids.org/) at 100–200 cm depth (Pongmala et al., 2022) and 250 m spatial resolution.

The distance to provincial hospital was measured as the Euclidean distance from the commune centroid to the respective provincial hospital. The GPS coordinates of the provincial hospitals were determined using Google Earth Pro version 7.3.6.9345 (Google, CA, USA 2022) and coded as hub points in QGIS. Distance to waterways was derived from the OSM 100 m raster (https://www.worldpop.org/). These data were aggregated to the commune level using zonal statistics (mean). To develop a variable representing the percentage of commune dedicated to agriculture a land cover/land use classification for Vietnam was used (https://www.landcovermapping.org/en/landcover/). The sum of classified cells was calculated for each commune using zonal statistics (count). All cells that were not classified as “cropland” were masked and the sum of cropland cells were calculated for each commune; the resulting percentage of cropland per commune was calculated. Human population counts were used to estimate population density per km^2^ in a commune; 100 m rasters of human count data (https://www.worldpop.org/) were aggregated to the commune level (zonal statistics–sum) and the sum of human population was divided by the area per km^2^ for that commune.

### 2.7 Eigenvector‐Based Spatial Filtering and Logistic Regression

To evaluate the relationship between commune‐level seropositivity and environmental factors, logistic regression models were developed in R. Given the spatial structure in both environmental and serological sampling data, a spatial filtering approach using Moran's eigenvector maps (MEMs) was implemented prior to variable selection (Dray et al., 2006). This process ensures that the potential influence of environmental factors is not overstated simply due to positive autocorrelation.

To prepare for modeling, continuous explanatory variables were transformed as necessary via square root, log, and logit to improve skew prior to scaling all variables (Borcard et al., 2011). Binary presence/absence of liberal and conservative seropositivity were used as univariate response variables.

Spatial filtering was conducted using the adespatial, sp, and spdep packages (Bivand et al., 2013; Bivand & Wong, 2018; Dray et al., 2023). A spatial weights matrix (SWM) was created consisting of a K‐nearest neighbors (*k* = 4; KNN) connectivity matrix weighted by inverse distance (1–distance/maximum distance). This distance decay relationship was incorporated based on the assumption that neighboring communes closer together would be more likely to have similar values than those farther apart (Taylor, 1983). Moran's eigenvector maps (MEMs; *n*–1) were calculated based on the SWM to describe spatial autocorrelation in sampling locations. The Moran's coefficients of positive eigenvectors were tested for significance, then forward‐selected against each response variable using the double‐stopping criterion described by Blanchet et al. (2008), which helps to reduce overfitting in the presence of many significant MEMs. Selected MEMs were then used as null spatial models and Pearson residuals were tested for significance of the Moran's I statistic to ensure autocorrelation was accounted for before proceeding (Bivand & Wong, 2018; Dray et al., 2023).

Forward stepwise selection was performed separately for liberal and conservative seropositivity using the MASS package, starting with null spatial models and adding environmental predictors (Venables & Ripley, 2002). Afterward, selected predictors were manually dropped if ΔAIC <2, indicating the model improvement was insufficient to justify the added complexity. Environmental variables were tested for spatial autocorrelation, and final model Pearson residuals were tested to confirm this autocorrelation was successfully described by the selected MEMs. Stability and performance of liberal and conservative models were then evaluated by calculating the events per variable (EPV; number of explanatory variables divided by the number of response presences), log odds ratios (OR), and validation through bootstrapped resampling (*n* = 999 repetitions) using the rms package (Harrell, 2026).

More detailed analysis methods are provided in the (Text S1 in Supporting Information S1), including *R* functions used for each step.

### 2.8 Spatial Smoothing and Spatial Hotspot Detection of Seropositivity

While the logistic regression approach was used to relate environmental conditions to potential *B. pseudomallei* exposure (in a presence/absence framework), we also wanted to examine potential spatial clusters of high rates of exposure. For this, we employed a rate smoothing and local cluster detection approach. Spatial smoothing of liberal and conservative commune‐level seropositivity rates was performed. The proportion of IgG‐seropositive patients per commune was calculated for both liberal and conservative cutoffs. Spatial Bayes rate smoothing (SBS) was performed in GeoDa version 1.20.0 to shrink the variance of the rate estimates using the K‐nearest neighbor (*n* = 4; KNN) with a distance decay function written as 1/distance (power = 1). The resulting distributions of the smoothed proportion of patients IgG seropositive, following liberal and conservative definitions, per commune were mapped using QGIS (Anselin, Lozano, & Koschinsky, 2006).

To determine if there was spatial dependence in the smoothed liberal and conservative commune seropositivity, Local Moran's I was run in GeoDa version 1.20.0 (Anselin, Syabri, & Kho, 2006) and resulting clusters were mapped in QGIS. The formula for this statistic is described as:

$$I_{i}=Z_{i}\sum W_{ij}Z_{j}$$

where *I*_*i*_ is the statistic for a commune *i*, Z_*i*_ is the difference between the proportional value of patients IgG seropositive at *i* and the mean proportional value of patients IgG seropositive for the study area, *W*_*ij*_ is the weights matrix (KNN4 with distance decay), and Z_*j*_ is the difference between the proportional value of patients IgG seropositive at *j* and the mean proportional value of patients IgG seropositive for the study area. This analysis was performed with 999 iterations and a significance level of *p* < 0.05.

## 3 Results

### 3.1 Sampling Demographics

This study tested human sera from 2,440 febrile patients that presented at provincial hospitals between 2020–2023. A STROBE guided flow diagram of inclusions/exclusions and sampling populations are detailed in Figure S7 in Supporting Information S1. Population characteristics for the study are presented in Table 1. The median age for the study area was 39 years old. The gender ratios were balanced between men and women in the overall study and throughout individual provinces; this varied in Son La province where there is approximately twice as many women patients than men. A small subset (14/2,440; 0.57%) of patients were missing gender information. Most patients fall between 20 and 70 years old (∼82%), however, fringe age groups such as young children, infants, and the elderly were somewhat represented across the study. Provinces with lower patient counts (Cao Bang; *n* = 95, Son La; *n* = 204) have no/minimal representation in these age groups.

**Table 1:** Demographic Characteristics of the Sampling Population

	Study area	Cao Bang	Dien Bien	Ha Giang	Lai Chau	Lao Cai	Son La
Age	*n* = 2,440	*n* = 95	*n* = 673	*n* = 243	*n* = 736	*n* = 489	*n* = 204
Median (IQR)	39 (28)	35 (24.3)	40 (29)	31 (34.3)	34 (28)	46.5 (29)	43 (23)
Age group							
	n (%)	n (%)	n (%)	n (%)	n (%)	n (%)	n (%)
<1	26 (1.07)	0 (0)	4 (0.59)	6 (2.47)	13 (1.77)	1 (0.2)	1 (0.49)
1–4	51 (2.09)	0 (0)	1 (0.15)	30 (12.35)	19 (2.58)	0 (0)	1 (0.49)
5–9	32 (1.31)	2 (2.11)	0 (0)	17 (7)	13 (1.77)	0 (0)	0 (0)
10–19	231 (9.47)	14 (14.74)	55 (8.17)	28 (11.52)	106 (14.4)	21 (4.29)	7 (3.43)
20–29	405 (16.6)	9 (9.47)	130 (19.32)	30 (12.35)	130 (17.66)	75 (15.34)	31 (15.2)
30–39	489 (20.04)	27 (28.42)	133 (19.76)	44 (18.11)	153 (20.79)	86 (17.59)	46 (22.55)
40–49	378 (15.49)	14 (14.74)	95 (14.12)	25 (10.29)	111 (15.08)	83 (16.97)	50 (24.51)
50–59	408 (16.72)	15 (15.79)	113 (16.79)	36 (14.81)	111 (15.08)	93 (19.02)	40 (19.61)
60–69	313 (12.83)	9 (9.47)	109 (16.2)	26 (10.7)	55 (7.47)	88 (18)	26 (12.75)
70–79	75 (3.07)	3 (3.16)	23 (3.42)	1 (0.41)	16 (2.17)	31 (6.34)	1 (0.49)
80+	32 (1.31)	1 (1.05)	10 (1.49)	0 (0)	9 (1.22)	11 (2.25)	1 (0.49)
Gender							
Men	1,213 (49.71)	54 (56.84)	327 (48.59)	117 (48.15)	379 (51.49)	271 (55.42)	65 (31.86)
Women	1,213 (49.71)	41 (43.16)	342 (50.82)	126 (51.85)	351 (47.69)	218 (44.58)	135 (66.18)
Missing	14 (0.57)	0 (0)	4 (0.59)	0 (0)	0 (0)	0 (0)	4 (1.96)

### 3.2 Exploratory Blood Culturing and Phylogenetics

**Figure 2:**
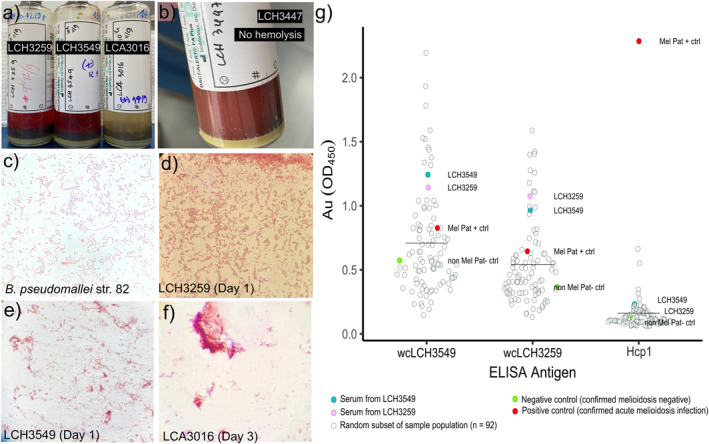
Blood culturing and resulting Gram stain imagery from blood bottle isolates in study. Three of the four blood cultures showed hemolysis (a) and no signs of hemolysis were observed in the fourth (b). A Gram stain image of *B. pseudomallei* Bp82 strain as a positive control (c). Gram stain imagery of the three samples showing hemolysis in blood culture (d, e, f). Absorbance values (OD450) of ELISA assays using whole cell lysate from two patient isolates (LCH3549 and LCH3259) and Hcp1 as antigens (g). Mean values for the assay sample population (*n* = 96) are shown as a black line. Absorbance values from sera patient samples, LCH3549 and LCH3259, are colored in teal and pink, respectively. Absorbance values from a known melioidosis negative patient are shown in green. Absorbance values from a patient with a confirmed active melioidosis infection are shown in red.

Blood culturing of a subset of initially seroreactive patients (*n* = 14) was performed and growth was observed in 4 bottles following a maximum 96 hr incubation (Figures 2a and 2b). Hemolytic activity was observed in 3 of 4 cultures (Figure 2a). Gram staining of the three cultures showing hemolytic activity was performed and characteristics typical for *B. pseudomallei* were observed (Figures 2d–2f); a reference Gram stain is provided for *B. pseudomallei* Bp82 (Figure 2c). However, it was determined the four bacterial isolates recovered were negative for *B. pseudomallei* based on TTS1 qPCR prompting whole genome sequencing to confirm the identity of these cultures.

**Figure 3:**
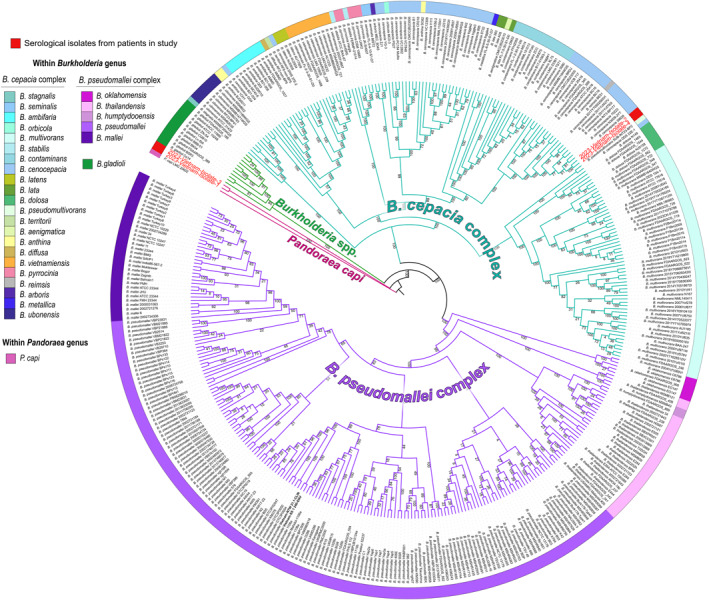
Maximum‐likelihood phylogeny inferred using PhAME (RAxML) based on core genome SNPs of blood‐bottle isolates and genetic neighbors from *Burkholderia* spp., *Burkholderia pseudomallei* complex, *Burkholderia cepacia* complex, and *Pandoraea capi* species. Strains that were isolated from patients in this study and whole genome sequenced are shown in red bold text and their outer ring is colored red. Branches are colored by genus or genus complex (pink = *Pandoraea* genus, green = *Burkholderia* genus, teal = BCC, purple = BPC). Species within each complex (or genus) are listed in the legend with colors correlating to the outer ring of the tree. The *Pandoraea capi* reference genome was used as outgroup taxa for rooting.

Illumina sequencing produced upwards of 100X coverage for each isolate utilizing an average length of 8 Mbp (in reference to *Burkholderia* genus and its variability is size). Assembled genomes for samples LCA3016 and LCH3447 were identified as *Pandoraea capi* (Table S4 in Supporting Information S1). Genomes from LCH3549 and LCH3259 (2023‐Vietnam‐Isolate‐4 and 2023‐Vietnam‐Isolate‐2) were partially identified as *Burkholderia reimsis* and *Burkholderia cenocepacia* (Table S4 in Supporting Information S1). A phylogenetic analysis confirmed that isolates LCA3016 and LCH3447 (2023‐Vietnam‐Isolate‐1 and 2023‐Vietnam‐Isolate‐3) were closely related the *P. capi* reference genome (Figure 3). The *B. gladioli* clade (*Burkholderia* spp., colored in green) fell between the *P. capi* tree leaves (colored in pink) and the greater BCC (colored in teal) (Figure 3). Isolates LCH3549 and LCH3259 fell within the greater BCC and more specifically, in the same sub‐clade as *B. reimsis* (colored in gray on the outer species ring), containing additional *B. cenocepacia* strains (Figure 3). Species within the BCC, a total of 23 species with reference quality genomes in NCBI, were interspersed on the phylogenetic tree (Figure 3). The BCC is closely related to species within the BPC (Figure 3). Within the *B. pseudomallei* complex, near‐neighbor species *B. oklahomensis*, *B. thailandensis*, and *B. humptydooensis*, are closer to the BPC strains than *B. pseudomallei* strains and the *B. mallei* strains (Figure 3).

Serological assays using whole cell lysate from the two isolates placed in the genus *Burkholderia* (LCH3549 and LCH3259) were used as a limited assessment for potential cross reactivity that may be occurring in our *B. pseudomallei* ELISA assays (OPS and Hcp1) (Figure 2g). The whole cell lysate assays both showed minimal reactivity (low absorbance values) to the melioidosis patient positive control (red circle) (Figure 2g). Repeated testing on the Hcp1 assay showed minimal reactivity to the LCH3549 and LCH3259 serum (blue and pink dots, respectively) and a strong reaction to the melioidosis positive control serum (red dots) (Figure 2g).

### 3.3 Seropositivity

**Figure 4:**
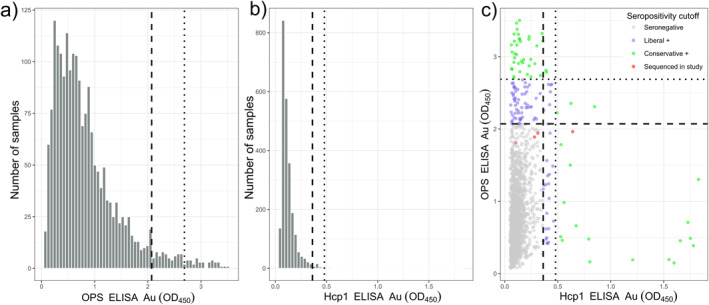
Distributions of OPS and Hcp1 IgG ELISA absorbance values. Histograms of OPS (a) and Hcp1 (b) absorbance values, with a hashed line representing ≥μ + 2σ and a dotted line representing ≥μ + 3σ for each population. A comparison of OPS and Hcp1 IgG ELISA with respective liberal and conservative cutoff values (hashed and dotted line, respectively) (c). The four sequenced samples are shown in red (c).

Two IgG ELISAs targeting past or cumulative exposure to *B. pseudomallei* were used in this study, OPS and Hcp1. Following our dual‐threshold definitions, a total of 149/2,440 (6.1%) individuals were determined as liberal seropositive, with either assay surpassing ≥*µ* + 2σ; and 61/2,440 (2.5%) individuals were defined as conservative seropositive, with either assay surpassing ≥*µ* + 3σ (Figure 4c, Table 2). Within assay, the OPS IgG ELISA was run on 1,979/2,440 patient samples (81%) (Figure 4a). Of these, 105/1,979 (5.3%) were considered liberal seropositive (≥*µ* + 2σ) surpassing 2.072 Au and 38/1,979 (1.9%) were considered conservative seropositive (≥*µ* + 3σ) surpassing 2.688 Au (Figure 4a). The Hcp1 IgG ELISA was run on all 2,440 patient samples (Figure 4b). Of these, 55/2,440 (2.3%) were considered liberal seropositive (≥*µ* + 2σ) surpassing 0.362 Au; and 23/2,440 (0.94%) were considered conservative (≥*µ* + 3σ) seropositive surpassing 0.479 Au (Figure 4b).

**Table 2:** Seropositivity by Demographic Groups (Gender, Age Groups, Province) for Liberal and Conservative Cutoffs

Demographic	Group	Seronegative	Seropositive	p‐value
Liberal		n (%)	n (%)	
n		2,291 (93.9)	149 (6.1)	
Gender (%)	Missing	13 (0.6)	1 (0.7)	0.689
	Men	1,144 (49.9)	69 (46.3)	
	Women	1,134 (49.5)	79 (53.0)	
Age group (%)	<1	25 (1.1)	1 (0.7)	0.651
	1–4	50 (2.2)	1 (0.7)	
	5–9	30 (1.3)	2 (1.3)	
	10–19	218 (9.5)	13 (8.7)	
	20–29	383 (16.7)	22 (14.8)	
	30–39	465 (20.3)	24 (16.1)	
	40–49	353 (15.4)	25 (16.8)	
	50–59	376 (16.4)	32 (21.5)	
	60–69	292 (12.7)	21 (14.1)	
	70–79	68 (3.0)	7 (4.7)	
	80+	31 (1.4)	1 (0.7)	
Province (%)	Cao Bang	94 (4.1)	2 (1.3)	<0.001
	Dien Bien	644 (28.1)	31 (20.8)	
	Ha Giang	230 (10.0)	10 (6.7)	
	Lai Chau	665 (29.0)	71 (47.7)	
	Lao Cai	462 (20.2)	26 (17.4)	
	Son La	196 (8.6)	9 (6.0)	
Conservative				
n		2,379 (97.5)	61 (2.5)	
Gender (%)	Missing	13 (0.5)	1 (1.6)	0.396
	Men	1,180 (49.6)	33 (54.1)	
	Women	1,186 (49.9)	27 (44.3)	
Age group (%)	<1	26 (1.1)	0 (0.0)	0.654
	1–4	50 (2.1)	1 (1.6)	
	5–9	31 (1.3)	1 (1.6)	
	10–19	227 (9.5)	4 (6.6)	
	20–29	397 (16.7)	8 (13.1)	
	30–39	476 (20.0)	13 (21.3)	
	40–49	371 (15.6)	7 (11.5)	
	50–59	392 (16.5)	16 (26.2)	
	60–69	303 (12.7)	10 (16.4)	
	70–79	74 (3.1)	1 (1.6)	
	80+	32 (1.3)	0 (0.0)	
Province (%)	Cao Bang	94 (4.0)	2 (3.3)	0.351
	Dien Bien	663 (27.9)	12 (19.7)	
	Ha Giang	233 (9.8)	7 (11.5)	
	Lai Chau	710 (29.8)	26 (42.6)	
	Lao Cai	478 (20.1)	10 (16.4)	
	Son La	201 (8.4)	4 (6.6)	

There were no significant differences observed between gender (*p* = 0.689 and *p* = 0.396) or age groups (*p* = 0.651 and *p* = 0.654). The provincial seropositivity rates varied significantly by province (*p* < 0.001) following the liberal seropositivity cutoff, with Lai Chau accounting for 47.7% of the seropositive patients. Following the conservative definition, Lai Chau still accounted for 42.6% of the seropositive cases but no significant difference between age groups was identified. Seropositivity by demographic groups for OPS and Hcp1 IgG ELISAs, following both liberal and conservative definitions, are provided in Table S5.

**Figure 5:**
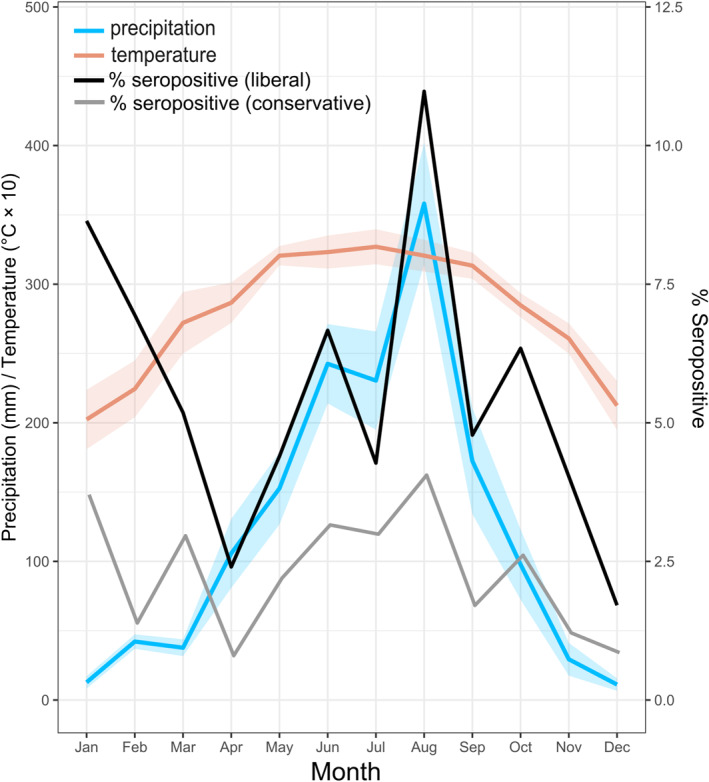
Monthly seropositivity rates throughout the study period. Average monthly precipitation (blue line) and maximum temperature (orange line) are shown as means (lines) with standard deviations (ribbons) across the six provinces for the 4‐year study period (2020–2022). Monthly seropositivity rates are shown using the liberal definition (≥mean + 2 SD; black) and the conservative definition (≥mean + 3 SD; gray). Seropositivity rates correspond to the right *y*‐axis.

An exploratory seasonal analysis using patient admission dates and seropositivity is presented in Figure 5. The proportional value of patients IgG seropositive to *B. pseudomallei* OPS or Hcp1 ELISA assays, following the liberal and conservative definition, was highest in August (11% and 4.1%). August also has the highest reported average annual precipitation during the study period (Figure 5).

### 3.4 Commune‐Level Environmental and Geographic Correlates

A total of 178 positive MEMs were created based on the SWM, of which 162 were significant through Monte‐Carlo testing of Moran's I with 999 permutations (Table S6). All transformed, scaled explanatory variables contained significant positive autocorrelation (Table S7 in Supporting Information S1). Global Moran's *I* tests during MEM selection were significant (liberal: *I* = 0.181, *p* = 0.0002*; conservative: *I* = 0.192, *p* = 0.0005*), indicating spatial autocorrelation was present within the response data and MEMs should be incorporated in subsequent analyses. Forward selection identified nine MEMs each for liberal and conservative seropositivity, where four were common to both models (Table S8 in Supporting Information S1). Pearson residuals of null spatial models containing these selected MEMs demonstrated that spatial filtering was successful, as neither model contained significant autocorrelation relative to the expected Moran's *I* value (*I* = −0.002) if residuals were randomly distributed (liberal: *I* = 0.010, *p* = 0.336; conservative: *I* = −0.014, *p* = 0.657).

Forward selection of explanatory variables for identified three predictors of liberal seropositivity: accumulated precipitation 2020–2022, organic Carbon, and distance to hospital; and three predictors of conservative seropositivity: distance to waterway, soil pH, and accumulated precipitation. However, after dropping variables that decreased model AIC by less than two, final models were as follows: liberal seropositivity was predicted by nine MEMs, accumulated precipitation, and organic Carbon; conservative seropositivity was predicted by nine MEMs, distance to waterway, and soil pH (Table 3). Pearson residuals showed that spatial autocorrelation was managed in both final models (liberal: *I* = −0.003, *p* = 0.516; conservative: *I* = −0.033, *p* = 0.858).

**Table 3:** Output of Selected Generalized Linear Models Using Both Liberal and Conservative Seropositivity Cutoffs

Term	Estimate	Standard error	z‐score	*p*‐value
Liberal Seropositivity				
Intercept	−1.619	0.146	−11.065	<0.001*
MEM17	−0.355	0.143	−2.485	0.013*
MEM6	0.649	0.162	4.016	0.000*
MEM144	0.426	0.122	3.486	0.000*
MEM135	−0.347	0.117	−2.967	0.003*
MEM1	−0.552	0.171	−3.220	0.001*
MEM25	0.352	0.129	2.731	0.006*
MEM7	−0.189	0.127	−1.483	0.138
MEM22	−0.296	0.131	−2.262	0.024*
MEM2	−0.333	0.237	−1.401	0.161
Average Accumulated Precipitation 2020–22 (mm)	0.526	0.173	3.037	0.002*
Organic Carbon (dg/kg)	−0.319	0.119	−2.681	0.007*
Conservative Seropositivity				
Intercept	−2.841	0.238	−11.959	<0.001*
MEM25	0.611	0.217	2.814	0.005*
MEM56	−0.686	0.169	−4.069	0.000*
MEM22	−0.552	0.172	−3.213	0.001*
MEM6	0.320	0.180	1.774	0.076
MEM150	−0.493	0.163	−3.032	0.002*
MEM144	0.538	0.170	3.164	0.002*
MEM13	−0.479	0.176	−2.719	0.007*
MEM8	0.251	0.174	1.447	0.148
MEM155	0.428	0.150	2.856	0.004*
Distance to waterway (km)	−0.427	0.201	−2.128	0.033*
Soil pH *10	−0.286	0.128	−2.237	0.025*

*Note.* Spatial autocorrelation components are abbreviated with the Moran's eigenvector map (MEM) number. See (Text S1 in Supporting Information S1) for further information on MEMs. * indicates statistical significance.

The liberal model had an EPV of 9.27 (11 variables/102 presences) and the conservative model had an EPV of 4.73 (11 variables/56 presences). Log odds ratios with 95% confidence intervals for environmental predictors in the liberal model were as follows: precipitation (1.69, 1.21–2.39) and organic Carbon (0.73, 0.58–0.92); and in the conservative model: distance to water (0.65, 0.44–0.96) and soil pH (0.75, 0.59–0.97). Apparent and optimism‐corrected model statistics are presented in Table S9 in Supporting Information S1. Although bootstrapping revealed modest overfitting, model discrimination remained high after optimism‐correction, thereby demonstrating stability in both models. Overall, the conservative model displayed greater predictive performance despite having a lower EPV than the liberal model.

Expanded results and model outputs are available in the, including Table S6, Text S2 and Tables S7–S9 in Supporting Information S1.

### 3.5 Spatial Smoothing and Hotspot Detection of Seropositivity

**Figure 6:**
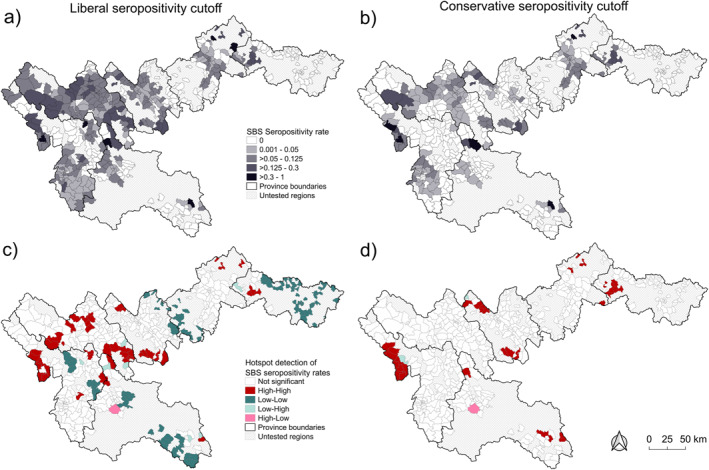
Distribution (a) and spatial clustering (b) of Spatial Bayes smoothed (SBS) liberal and conservative seropositivity rates (%) in tested communes in Northernmost Vietnam. Untested regions are shown in hatch markings. Communes with the highest SBS seropositivity (%) are shown in black (a). High‐High clusters are show in dark red with low‐low clusters shown in dark teal (b). Outliers, low‐high and high‐low clusters are shown in light teal and light red, respectively (b).

Spatial smoothing of commune‐level seropositivity rates following the liberal and conservative definitions showed a widespread spatial distribution across the northernmost provinces of Vietnam (Figures 6a and 6b). Much of the distribution was concentrated in the western portion of the study area, encompassing Dien Bien, Lai Chau, and Lao Cai provinces. Limited sampling coverage occurred in Son La and Cao Bang provinces; and no commune‐level seroreactivity was identified in the eastern portion of Cao Bang in tested areas (Figures 6a and 6b). The Local Moran's *I* statistic identified significant spatial clustering in commune‐level SBS seropositivity across the study area, with the greatest number of High‐High clusters (hotspots) present in the western portion of the study area, specifically Lai Chau and Dien Bien provinces (Figures 6c and 6d). In the spatial clustering analysis using the liberal seropositivity cutoff Low‐Low clusters (cold spots) were primarily distributed in the northern and southern portion of the study area: Cao Bang and Son La province, respectively (Figure 6c). No cold spots were identified in the spatial clustering analysis derived from the conservative cutoff (Figure 6d). Furthermore, fewer hotspots were detected in the conservative analysis; and all hotspots detected in Lai Chau were no longer significant in the conservative analysis (Figure 6d).

## 4 Discussion

This hospital‐based cross‐sectional study reports on elevated *B. pseudomallei* serological responses in an opportunistic febrile hospital population across the six northernmost provinces of Vietnam. While the primary target was *B. pseudomallei*, all four blood culture isolates recovered from patients with initially elevated serological responses were negative on qPCR for *B. pseudomallei*, prompting whole genome sequencing for further characterization. Phylogenetic analysis revealed that isolates from the blood culture had close genetic relationships to *B. pseudomallei* and are considered opportunistic pathogens capable of causing disease (Costello et al., 2011; Kruis et al., 2023; Vandamme et al., 1997). Two of the isolated species were partially identified as members of the BCC (Vandamme & Dawyndt, 2011). The remaining two isolates were identified as *Pandoraea capi*.

These findings are clinically significant. Bacteria in the BCC have adapted to survive in microbially competitive aqueous environments containing antimicrobials and disinfectants (Tavares et al., 2020), leading to contamination and subsequent nosocomial infections (Coenye & LiPuma, 2003; Jimenez, 2001; Pankhurst & Philpott‐Howard, 1996). Multiple cases of hospital acquired infections from BCC opportunistic bacteria were reported in Vietnam in 2024 (Nguyen‐Dang et al., 2024)–one presenting with a multiple antimicrobial resistant phenotype (*B. dolosa*) (Nguyen et al., 2024). *Pandoraea* spp. have shown virulence akin to *Burkholderia cenocepacia* (a species in BCC) in *vivo* models using *Galleria mellonella* (wax moth) larvae (Costello et al., 2011). Similar to *B. pseudomallei*, species in the BCC and *P. capi* have developed high resistance to antibiotics and many antiseptics (Nzula et al., 2002), making successful treatment and recovery challenging. These species also may occupy ecological niches similar to *B. pseudomallei*, suggesting similar exposure risks through soil and water (Compant et al., 2008).

Prior to this study, the northernmost provinces of Vietnam had little to no known history of melioidosis or *B. pseudomallei*. In contrast, a high disease burden is documented throughout Vietnam's central region, and the recent reports of pediatric mortalities have emerged in the north, suggesting the disease is present in lesser sampled regions. Here, elevated serological responses to *B. pseudomallei* were identified throughout the northernmost border provinces following liberal and conservative cutoffs (6.1% and 2.5% respectively). No significant associations to seropositivity were identified by gender or age of febrile patients in either cutoff.

Our findings are aligned with established epidemiological patterns regarding seasonality. The highest recorded rainfall was in August, correlating with a peak in *B. pseudomallei* seroreactivity rates in admitted febrile patients. It should be noted that in this purely exploratory examination IgG reactivity can remain for >1 year and does not necessarily imply active infections at time of admission. However, at the commune‐scale, the presence of seroreactive patients was correlated with higher average rainfall and proximity to major waterways. These findings align with the current hypothesis that *B. pseudomallei* is mobilized from the soil and plant rhizosphere during rainfall or monsoon events, with rising water levels cycling *B. pseudomallei* from deep in the soil column toward the surface (B. Currie et al., 2001; Inglis et al., 2001). Repeated monsoon events can further saturate the moist soil, raising the water table to the ground surface, causing flooding (Cheng & Currie, 2005; B. J. Currie et al., 2004). This movement to the soil surface or into water sources can lead to increased risk for environmental exposure–resulting in transmission through cuts or abrasions (B. J. Currie & Jacups, 2003). Potential aerosol or droplet transmission during these events has also been suggested, however, limited evidence support natural aerosolization of *B. pseudomallei* and subsequent human infections (P.‐S. Chen et al., 2015; B. J. Currie & Jacups, 2003). In other endemic regions, an increase in human melioidosis cases was observed following extreme weather events, such as heavy monsoon rains or tropical cyclones (Cheng et al., 2006; Kaestli et al., 2016; Merritt & Inglis, 2017).

The persistence of *B. pseudomallei* in the environment is commonly associated with low‐nutrient high‐moisture acidic soils. In this study, communes with seropositive patients had significantly lower median carbon contents and more acidic soil. Prior studies in nearby Laos PDR, Thailand, and Northern Australia, found similar associations to these soil conditions (Baker et al., 2015; Manivanh et al., 2017; Ngamsang et al., 2015). It has been suggested that *B. pseudomallei* may be outcompeted in nutrient rich soils as they may have higher biotic stressors due to increased competition for nutrients and abiotic substrates (Maier et al., 2009). A prior laboratory‐based study determined that optimal pH conditions for the growth of *B. pseudomallei* ranged between 6.5 and 7.5 (Y. S. Chen et al., 2003) and further studies utilizing soil from endemic regions in Thailand showed growth between pH 5–7,with significant decreases in bacterial growth above pH 8 (Wang‐ngarm et al., 2014). This study reported average soil pH values throughout the study area being 5.38 with presence communes being significantly more acidic than absence communes.

Widespread spatial patterns of *B. pseudomallei* seropositivity were identified in this study. Following the liberal seropositivity cutoff, provincial seropositivity rates differed significantly (*p* < 0.001) with Lai Chau province accounting for 47.7% of seropositive patients. At the commune‐scale, hotspots of elevated seroreactive cases were primarily identified in Lai Chau province. Notably, hotspot communes were identified in all six provinces, and many were not proximate to provincial hospitals. This indicates that these regions, despite potentially lower sampling due to accessibility, had elevated serological responses. In these northernmost Vietnam provinces, accessible healthcare can be limited (Thuong et al., 2022) and adequate communication with minority ethnic groups, with varying languages and cultural practices, can prove challenging (Målqvist et al., 2013; McKinn et al., 2017). Similar findings regarding high melioidosis prevalence in Australian rural regions, also comprised of mostly minority ethnic groups, have been established previously (Ashdown & Guard, 1984; Savelkoel et al., 2022). These findings suggest that an improvement in local and regional public health surveillance is required to elucidate the true underlying burden, particularly in areas of rural Vietnam. Studies targeting patients treated in local or regional health facilities within the hotspots are also suggested.

### 4.1 Limitations

Prior studies have noted challenges associated with successful blood culturing of *B. pseudomallei*, as the infection must be bacteremic having progressed into the bloodstream, rather than localized (Maze et al., 2020). In this study, blood culturing was an opportunistic, exploratory effort limited by available sample volumes and indirect inoculation methods, which differ from standard clinical protocols that utilize larger volumes to maximize sensitivity. Due to these limitations, the discovery of these organisms in blood culture should not be considered etiologic agents of the patients' febrile illness. However, the recovery of *Burkholderia* near‐neighbors does provide clinically relevant data regarding the potential circulation of opportunistic pathogens in the region. These findings underscore the need for further research into the diversity and prevalence of *Burkholderia* and *Pandoraea* species that cause human disease, particularly alongside clinical outcomes.

While this study lacked a comprehensive local control bank to rule out cross reactivity with *Pandoraea* spp. and BPC species, the use of OPS and Hcp1 as antigens has been assessed in previous studies. In Pumpuang et al. (2017), Hcp1 and OPS ELISAs demonstrated high specificity (98%–100% and 94%–100%, respectively) against other relevant pathogens, including scrub typhus, leptospirosis, and tuberculosis. Furthermore, our limited internal assessment of serum from two patients with blood‐cultured BPC organisms found negligible reactivity to *B. pseudomallei* antigens. Repeated testing of serum from two of the four isolates found that Hcp1 serological response was also lower than initially determined and the use of serum controls from known melioidosis negative and positive patients further confirmed that the blood‐cultured patients were not actively infected with *B. pseudomallei.* Notably, one *P*. *capi* isolate was recovered from a patient with detectable Hcp1 seropositivity; as IgG responses to *B. pseudomallei* can persist for over a year, this seropositivity may reflect prior exposure, rather than active infection, though non‐standard blood culturing conditions preclude definitive etiological attribution. Therefore, while further work is needed to determine the true sensitivity and specificity of these serological assays in this specific population, their use in the present study, in conjunction with an exploratory scope, is appropriate.

This study was opportunistic and based on convenience hospital‐based sampling. The findings of this study cannot be extrapolated to the general population as the sampling population consisted of febrile patients admitted to provincial hospitals. This group is unlikely to represent the general community in terms of age, comorbidities, or exposure risk. Furthermore, clinical outcomes of patients were not available in this study. Underlying spatial autocorrelation is present in the serological data sets, posing challenges for assessing environmental, clinical, and geographic correlations. Future targeted studies that are designed to estimate the true underlying burden and connections to human *B. pseudomallei* exposure for both ecological (persistence of pathogen) and behavioral/population correlates are needed.

## 5 Conclusions

This exploratory serological study reports on the past or cumulative exposure to *B. pseudomallei‐*related antigens in the northernmost provinces of Vietnam. While *B. pseudomallei* was not isolated from the small subset of blood culture isolates, the identification of BCC and *Pandoraea* spp. highlights a parallel risk: the circulation of opportunistic pathogens with a propensity for antimicrobial resistance which share similar ecological niches. Identification of these antibiotic‐resistant bacteria can cause difficulties in an area where infectious disease researchers and clinicians are working to improve disease surveillance and inform potential treatment in Northern Vietnam. The need for increased molecular surveillance required to differentiate closely related emerging antibiotic‐resistant infections should be communicated to public health workers and clinicians in the region.

Elevated antibody responses to *B. pseudomallei* reveal spatial patterns and hotspots of potential past exposures or infections in the region. Despite noted limitations, the observed clustering, seasonal association, and ecological correlations align with patterns reported in endemic regions of Thailand and Laos, suggesting the similar drivers of *B. pseudomallei* exposure may be operating in this region. This study begins to resolve a gap in knowledge on the distribution of *B. pseudomallei* in the country. Given the cross‐sectional, convenience‐based design, results should be interpreted as hypothesis‐generating rather than definitive estimates of exposure or disease incidence. Future studies that employ population‐based sampling, validated diagnostics, and longitudinal surveillance are needed to elucidate true melioidosis incidence.

## Inclusion in Global Research Statement

This study was conducted in partnership with the National Institute of Hygiene and Epidemiology (NIHE) and Vietnam National University. Data and sample collection was possible through collaborative efforts with provincial, district, and commune officials in Vietnam and the participation of local community members.

## Conflict of Interest

The authors declare no conflicts of interest relevant to this study.

## Supporting information

Supporting Information S1

Table S1

Table S2

Table S5

Table S6

## Data Availability

Disease data cannot be shared publicly due to IRB restrictions in Vietnam and the USA; reasonable data request can be considered via request to the Institute for Microbiology and Biotechnology, Vietnam National University (tttrung@vnu.edu.vn). All software and programs used in this study are open source: QGIS (https://qgis.org/en/site/), *R* (https://www.r‐project.org/), RStudio (https://rstudio.com/), GeoDa (https://geodacenter.github.io/), Inkscape (https://inkscape.org/), and Interactive Tree of Life (iTOL) (https://itol.embl.de/). Environmental and geographic data are publicly available and can be obtained through the links below: administrative boundaries (level 3, pre‐July 2025) from GADM (https://gadm.org/); soil properties (nitrogen, organic carbon, and pH at 100–200 cm depth, 250 m resolution) from SoilGrids (https://soilgrids.org/); monthly precipitation and soil moisture records (2020–2022, aggregated to 2021 means) from WorldClim 2 (http://worldclim.org/); GPS coordinates of provincial hospitals extracted from Google Earth Pro (v7.3.6.9345); distance to waterways derived from the OSM 100 m raster via WorldPop (https://www.worldpop.org/); cropland extent from the land‐cover/land‐use classification for Vietnam (https://www.landcovermapping.org/en/landcover/); and population density from WorldPop's 100 m raster (https://www.worldpop.org/).
