# Supplementary material for: Hospital‐Based Cross‐Sectional Study of Burkholderia pseudomallei Seroreactivity Among Febrile Patients in Northernmost Vietnam: Near‐Neighbor Bloodstream Isolates, Environmental Correlates, and Spatial Clustering
Source: Geohealth. 2026 May 30;10(6):e2025GH001606. doi: 10.1029/2025GH001606 (PMC13239667; doi:10.1029/2025GH001606)
Supplement: Supplementary file 1 — Supporting Information S1 [file GH2-10-e2025GH001606-s004.pdf]

GeoHealth

Supporting Information for

Hospital-based cross-sectional study of *Burkholderia pseudomallei* seroreactivity among febrile patients in northernmost Vietnam: near-neighbor bloodstream isolates, environmental correlates, and spatial clustering

Morgan C. Metrailer[1,2], Tran Thi Le Quyen[3], Khang Van Pham[4], Tan Minh Luong[4], Treenate Jiranantasak[1,2], Andrew P. Bluhm[1,2], Frank J. Tuozzo II[1,2], Thi Thu Ha Hoang[4], Minh Hoa Luong[4], Bich Ngoc Do[4], Thanh Hai Pham[4], Madison E.A. Harman[1,2], Michael H. Norris[2,5], Trinh Thanh Trung[3], Jason K. Blackburn*[1,2]

[1]Spatial Epidemiology & Ecology Research Laboratory, Department of Geography, University of Florida, Gainesville, Florida, United States of America

[2]Emerging Pathogens Institute, University of Florida, Gainesville, Florida, United States of America

[3]VNU - Institute for Microbiology and Biotechnology, Vietnam National University, Hanoi, Vietnam

[4]National Institute of Hygiene and Epidemiology, Hanoi, Vietnam

[5]Pathogen Analysis and Translational Health Group, School of Life Sciences, University of Hawai'i at Mānoa, Honolulu, Hawai'i, United States of America

Contents of this file

Text S1 to S2
Figures S1 to S6
Tables S3, S4, S7 to S9

Additional Supporting Information (Files uploaded separately)

Captions for Tables S1, S2, S5, S6

Text S1. Detailed methods on the data pre-processing and the logistic regression models with Moran's eigenvector spatial filtering.

To evaluate the relationship between commune-level seropositivity and environmental factors, logistic regression models were developed in R. Given the spatial structure in both environmental and sampling data, a spatial filtering approach using Moran's eigenvector maps (MEMs) was implemented prior to variable selection (Dray et al., 2006). This process ensures that the potential influence of environmental factors is not overstated simply due to positive autocorrelation.

Regressions were conducted separately for liberal and conservative seropositivity cutoffs, where the binary univariate response was presence or absence for each commune with at least one patient tested. Explanatory variables were examined for normality by calculating the coefficient of variation (CV) and skewness, then transformed as necessary via square root (i.e., organic Carbon [dg/kg], Nitrogen [cg/kg], and Euclidean distance to provincial hospital [km]), log (i.e., distance to waterway [km], and population density per km^2), and logit (percent cropland) (Borcard et al., 2011). Variables that did not need transformation included soil pH (pH *10), soil moisture (mm), and average accumulated precipitation from 2020 – 2022 (mm). All variables were then scaled to center the mean and standardize deviation (Borcard et al., 2011).

Spatial filtering was conducted using functions from the adespatial, sp, and spdep packages (Bivand et al., 2013; Bivand & Wong, 2018; Dray et al., 2023). A spatial weights matrix (SWM) was created using the listw.explore function's R Shiny application. The SWM was composed of a K-nearest neighbors (k = 4; KNN) connectivity matrix made with the chooseCN and coordinates functions and weighted by inverse distance (1 – distance / maximum distance) using the nbdists and nb2listw functions. This distance decay relationship was incorporated based on the assumption that neighboring communes closer together would be more likely to have similar values than those farther apart (Taylor, 1983). Using the SWM, spatial autocorrelation in sampling locations was calculated for total of n -1 orthogonal Moran's eigenvector maps (MEMs) using the mem function. The Moran's coefficients of positive eigenvectors were tested for significance with the moran.randtest function (n = 999 permutations). Environmental variables were similarly assessed for spatial autocorrelation through global (moran.randtest function) and two-sided tests (moranNP.randtest function), the latter of which separate positive and negative autocorrelation.

A subset of MEMs was forward selected against each response variable with the mem.select function, using the double-stopping criterion described by Blanchet et al. (2008) that incorporates both individual MEM significance ($\alpha < 0.05$) and prevents the model from exceeding the global adjusted R^2 to reduce overfitting. This procedure provides a global test of spatial autocorrelation in the response data, determining whether MEMs are necessary; if significant, selection proceeds. Selected MEMs were used in null spatial models via the glm function from base R's stats package. Then the moran.test function was used to test Pearson model residuals against the SWM for significance of the Moran's *I* statistic, where lack of significance shows that autocorrelation is not present, indicating it has been captured in the MEMs (Bivand & Wong, 2018; Dray et al., 2023).

Forward stepwise selection was performed on null models to add environmental predictors via the MASS package stepAIC function (Venables & Ripley, 2002). Afterward, selected predictors were manually dropped if ΔAIC < 2, indicating the model improvement was insufficient to justify the added complexity. Final model Pearson residuals were tested as previously described to confirm autocorrelation in the environmental predictors was successfully described by the MEMs.

Model stability was evaluated by calculating the events per variable (EPV; number of explanatory variables divided by the number of response presences), log odds ratios (OR), and bootstrapped resampling (n = 999 repetitions) using the lrm and validate functions of the rms package ; Harrell, 2026).

Text S2. Detailed results from the logistic regression models with Moran's eigenvector spatial filtering.

A total of 178 positive MEMs were created based on the SWM, of which 162 were significant through Monte-Carlo testing of Moran's I with 999 permutations (Table S6). All transformed, scaled explanatory variables contained significant positive autocorrelation (Table S7). Global Moran's I tests during MEM selection were significant (liberal: I = 0.181, p = 0.0002*; conservative: I = 0.192, p = 0.0005*), indicating spatial autocorrelation was present within the response data and MEMs should be incorporated in subsequent analyses. Forward selection of MEMs identified nine MEMs for the liberal seropositivity cutoff (MEM17, MEM6, MEM144, MEM135, MEM1, MEM25, MEM7, MEM22, and MEM2) and nine for the conservative cutoff (MEM25, MEM56, MEM22, MEM6, MEM150, MEM144, MEM13, MEM8, and MEM155), four of which were common to both models (Table S8). Pearson residuals of the null spatial models containing selected MEMs demonstrated that spatial filtering was successful, as neither model contained significant autocorrelation relative to the expected Moran's I value (I = -0.002) if residuals were randomly distributed (liberal: I = 0.010, p = 0.336; conservative: I = -0.014, p = 0.657).

Forward selection of explanatory variables against liberal seropositivity initially identified three environmental predictors (average accumulated precipitation 2020 – 2022, organic Carbon, and distance to hospital). However, after dropping variables that decreased model AIC by less than two, the final model of liberal seropositivity was predicted by nine MEMs, precipitation, and organic Carbon. For conservative seropositivity, forward selection identified three variables (distance to waterway, majority soil pH, and precipitation). Dropping low AIC terms resulted in a final model of conservative seropositivity predicted by nine MEMs, distance to waterway, and soil pH.

Pearson residuals showed that spatial autocorrelation was managed in both final models (liberal: I = -0.003, p = 0.516; conservative: I = -0.033, p = 0.858).

The EPV was 9.27 (11 variables / 102 presences) for the liberal model and 4.73 (11 variables / 56 presences) for the conservative model. Log odds ratios with 95% confidence intervals for environmental predictor variables were as follows for the liberal model: precipitation (1.69, 1.21-2.39) and organic Carbon (0.73, 0.58-0.92); and the

conservative model: distance to water (0.65, 0.44-0.96) and soil pH (0.75, 0.59-0.97). Apparent and optimism-corrected model statistics are in Table S9. Although bootstrapping revealed modest overfitting, model discrimination remained high after optimism-correction, thereby demonstrating stability in both models. Overall, the conservative model displayed greater predictive performance despite having a lower EPV value than the liberal model.

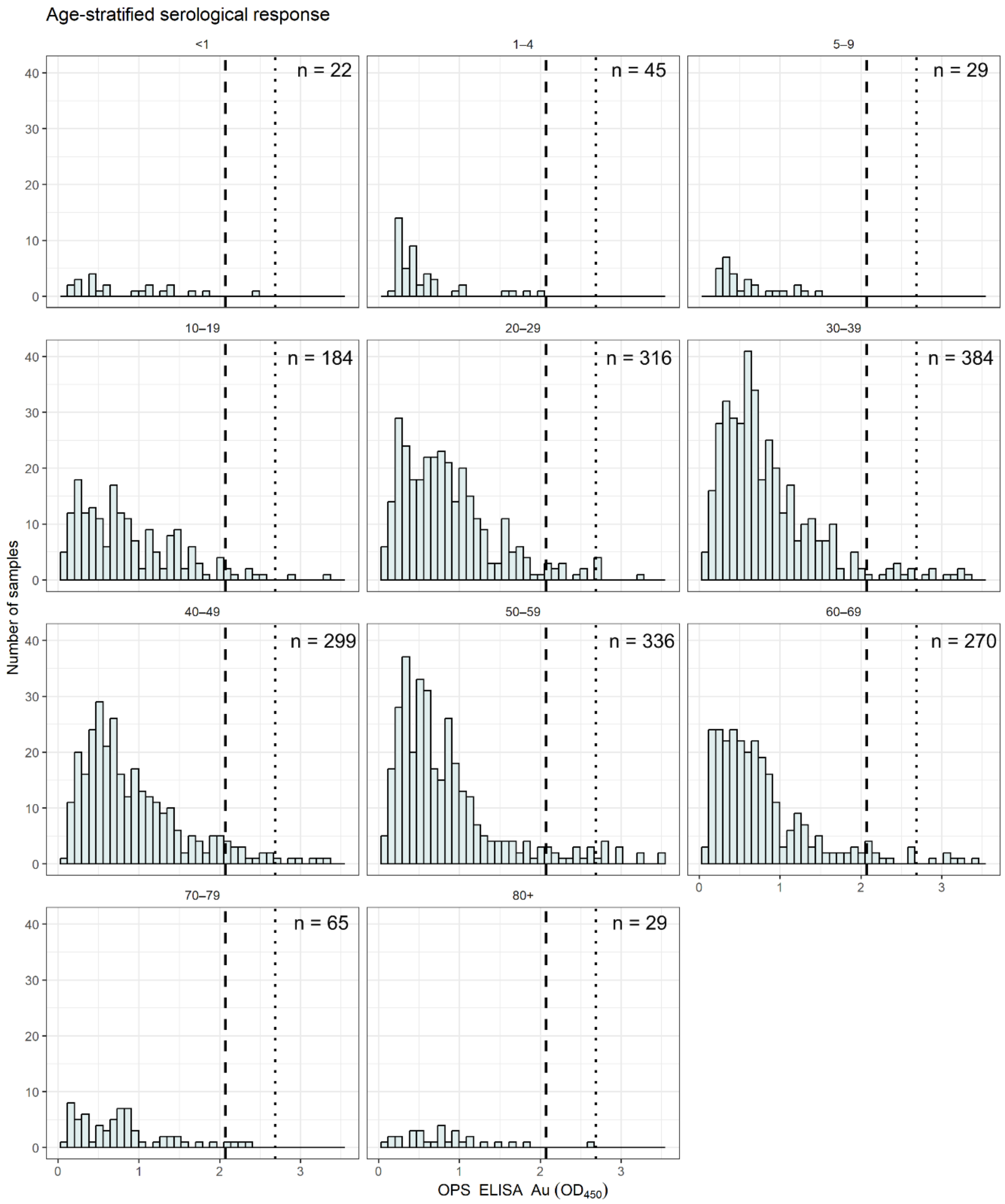

Figure S1. Histograms of OPS IgG ELISA absorbance values stratified by age group. Seropositivity cutoff values for the general population following liberal (≥ μ + 2σ; hashed line) and conservative (≥ μ + 3σ; dotted line) definitions are identified.

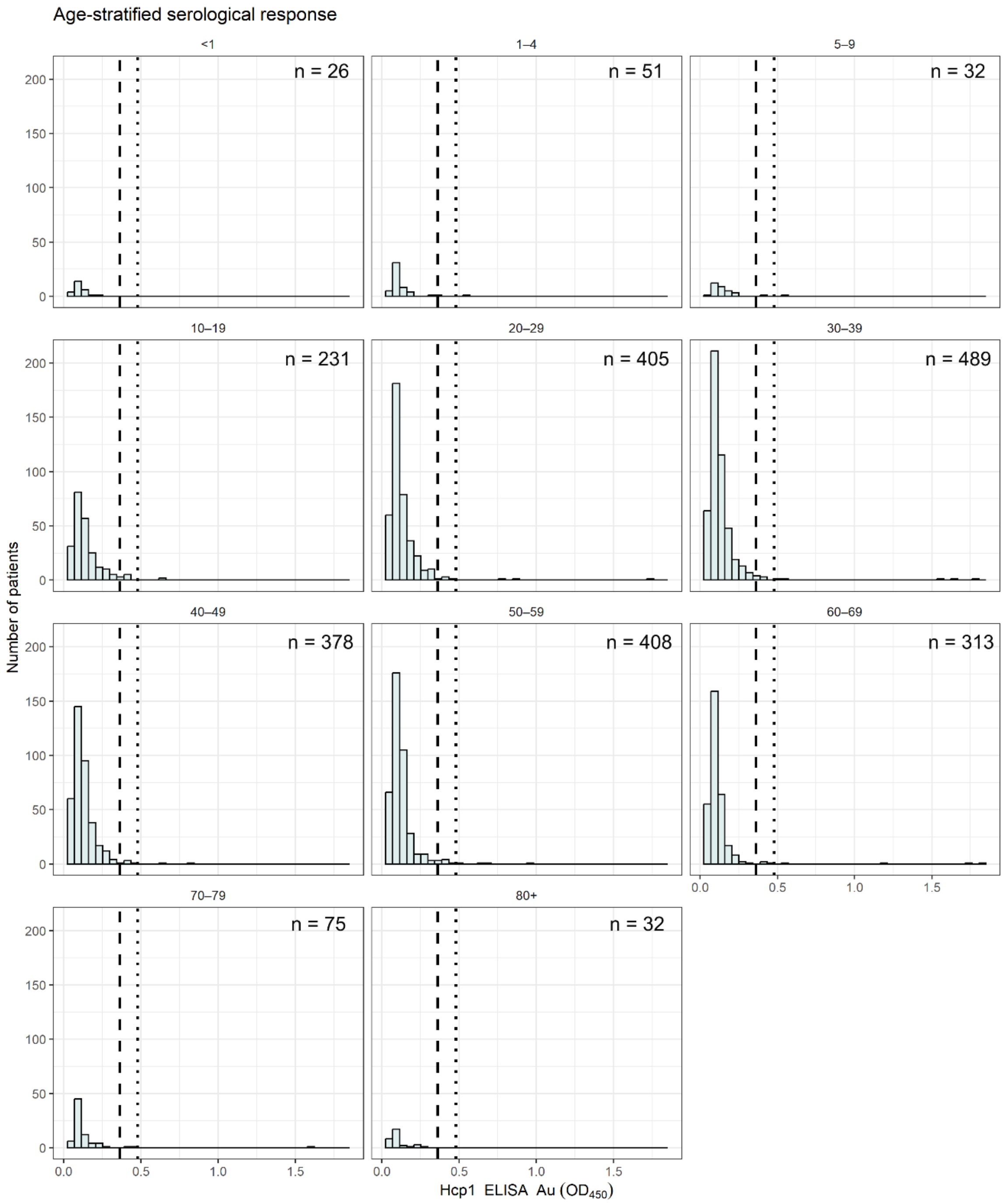

Figure S2. Histograms of Hcp1 IgG ELISA absorbance values stratified by age group. Seropositivity cutoff values for the general population following liberal (≥ μ + 2σ; hashed line) and conservative (≥ μ + 3σ; dotted line) definitions are identified.

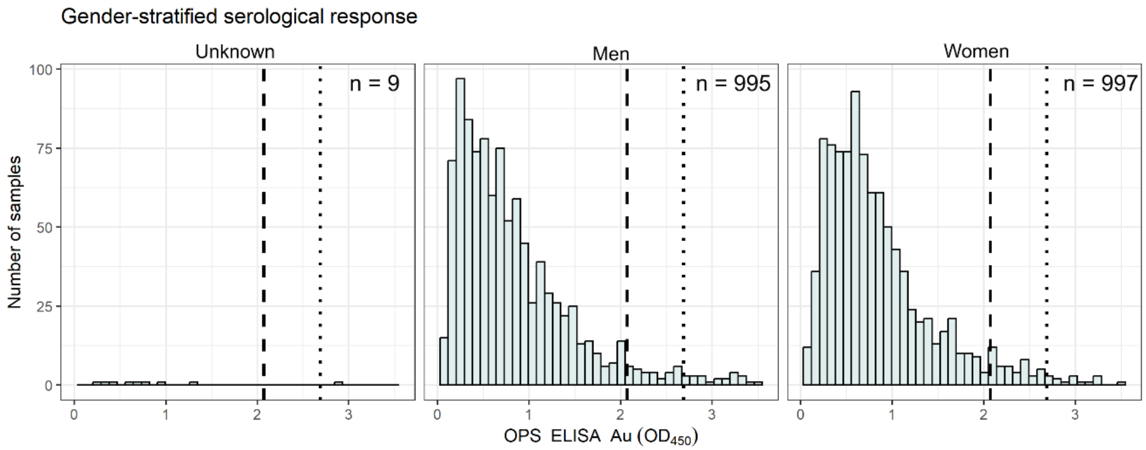

Figure S3. Histograms of OPS IgG ELISA absorbance values stratified by gender. Seropositivity cutoff values for the general population following liberal (≥ μ + 2σ; hashed line) and conservative (≥ μ + 3σ; dotted line) definitions are identified.

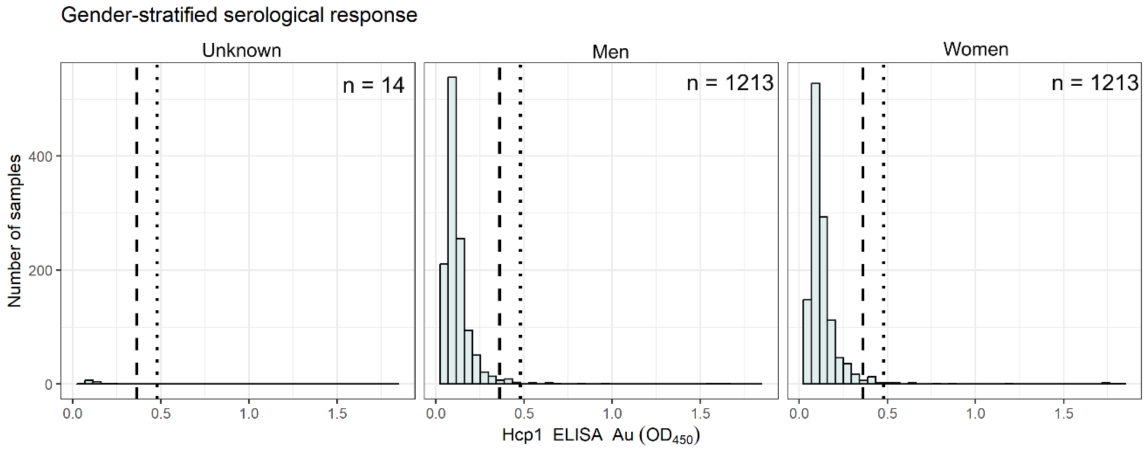

Figure S4. Histograms of Hcp1 IgG ELISA absorbance values stratified by gender. Seropositivity cutoff values for the general population following liberal (≥ μ + 2σ; hashed line) and conservative (≥ μ + 3σ; dotted line) definitions are identified.

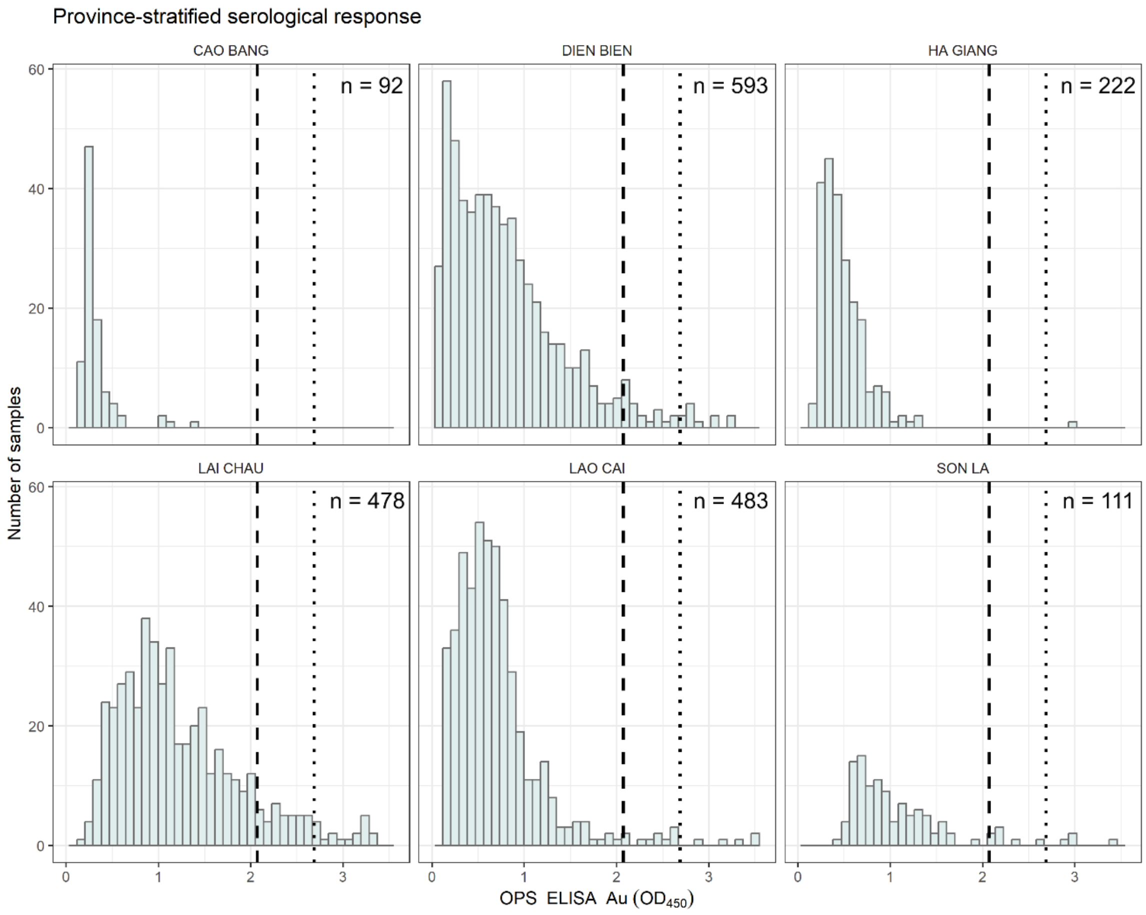

Figure S5. Histograms of OPS IgG ELISA absorbance values stratified by home province. Seropositivity cutoff values for the general population following liberal (≥ μ + 2σ; hashed line) and conservative (≥ μ + 3σ; dotted line) definitions are identified.

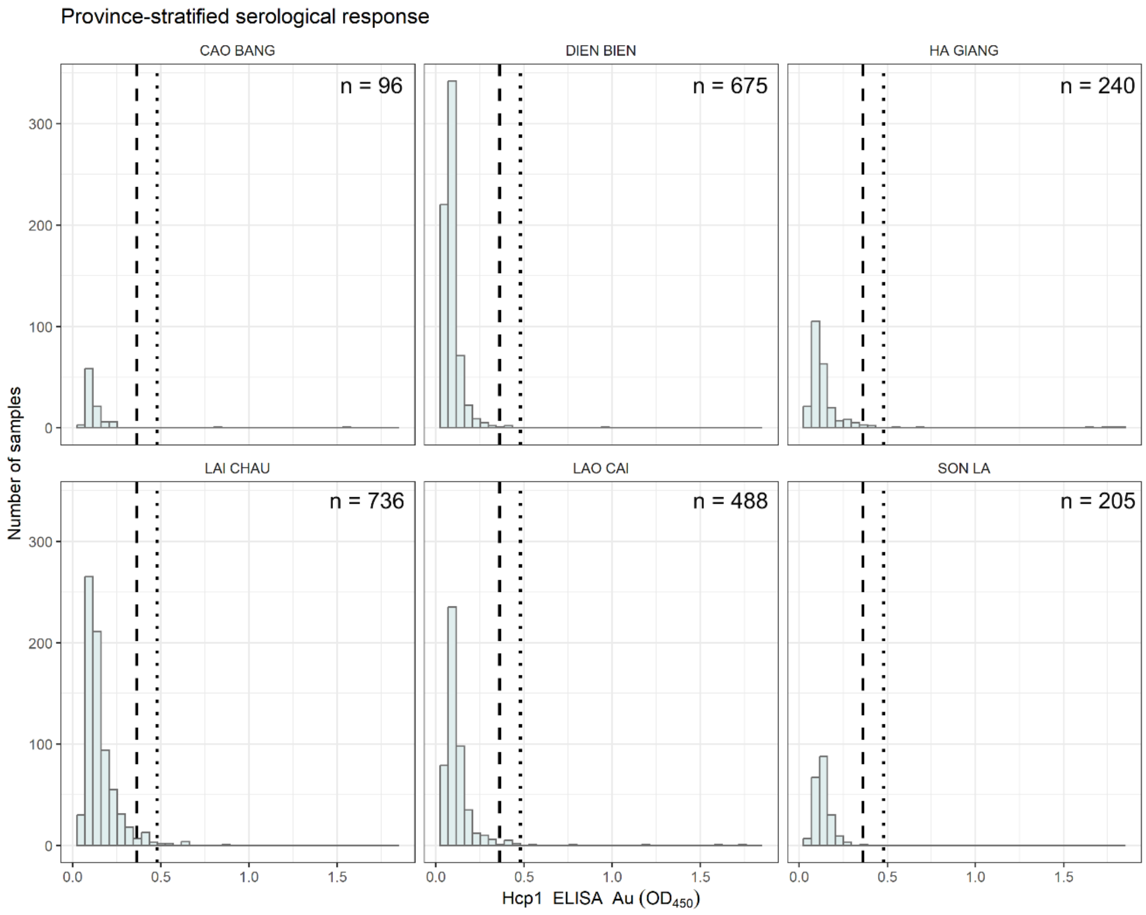

Figure S6. Histograms of Hcp1 IgG ELISA absorbance values stratified by home province. Seropositivity cutoff values for the general population following liberal (≥ μ + 2σ; hashed line) and conservative (≥ μ + 3σ; dotted line) definitions are identified.

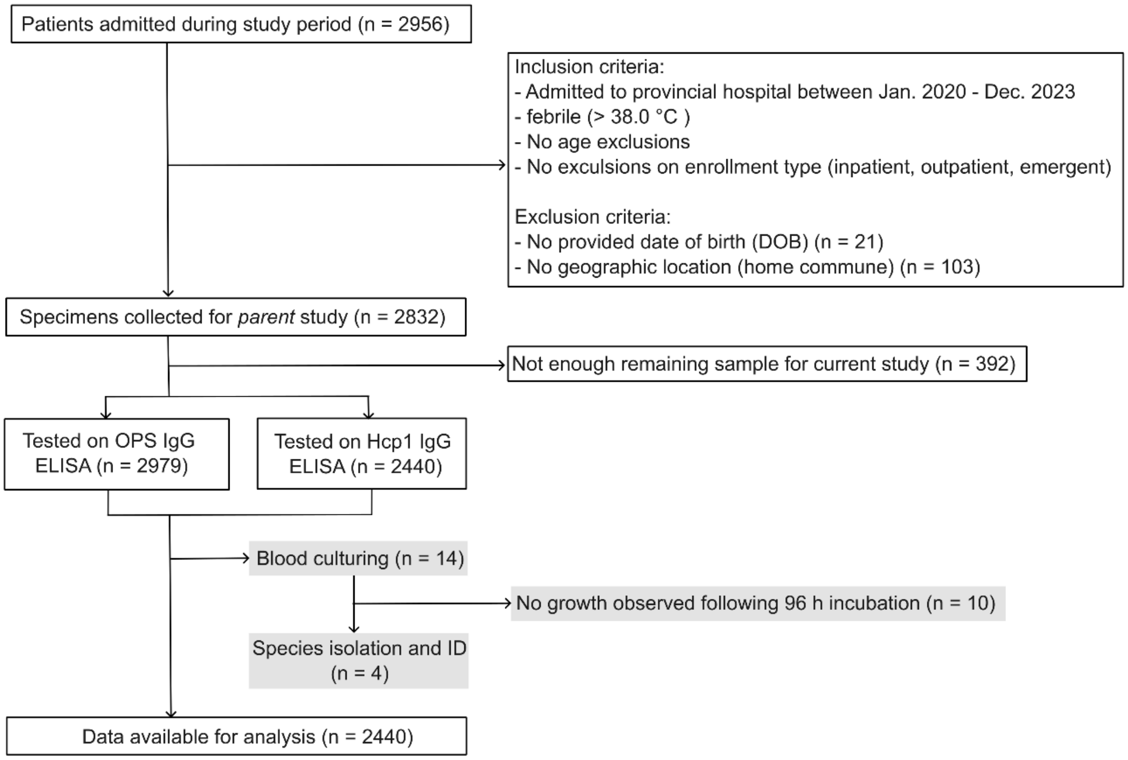

Figure S7. STROBE flow diagram detailing inclusion and exclusion criteria at each stage of the project.

Table S1. STROBE Checklist for cross-sectional studies.

Table S2. List of genomes used in this study. Genomes are identified by their phylogenetic tree ID and their genus, species, and species complex (if applicable) is detailed. The bacterial strain ID, NCBI accession number, and role as an ingroup or outgroup is denoted.

Table S3. Detailed information on the covariate data used in this study, including source, link, spatial resolution, and temporal range/period.

Variable	Source	Link	Spatial resolution	Temporal resolution
Nitrogen content (100-200 cm)	Soilgrids.org	https://soilgrids.org/	250 m	2020
Carbon content (100-200 cm)	Soilgrids.org	https://soilgrids.org/	250 m	2020

pH water *10 (100-200 cm)	Soilgrids.org	https://soilgrids.org/	250 m	2020
Accumulated precipitation	TerraClimate	https://www.climatologylab.org/terraclimate.html	4 km	2020-2022
Soil moisture	TerraClimate	https://www.climatologylab.org/terraclimate.html	4 km	2020-2022
Landcover - % crop	RLCMS – Land Cover Portal	https://www.landcovermapping.org/en/landcover/	30 m	2018
Unconstrained human population count	WorldPop	https://www.worldpop.org/datacatalog/	100 m	2020
OSM distance to waterway	WorldPop	https://www.worldpop.org/datacatalog/	100 m	2016

Table S4. Genus and species identification of bacterial strains isolated from patients in this study. PubMLST online tool was used to identify genus species and the respective percentage identity to that species.

Sample ID/Phylogenetic Tree ID	PubMLST Species ID	% identity
LCA3016/2023-Vietnam-Isolate-1	*Pandoraea capi*	100
LCH3259/2023-Vietnam-Isolate-2	*Burkholderia reimsis*	50
	Burkholderia cenocepacia	42
LCH3447/2023-Vietnam-Isolate-3	*Pandoraea capi*	100
LCH3549/2023-Vietnam-Isolate-4	*Burkholderia reimsis*	50
	Burkholderia cenocepacia	42

Table S5. Seropositivity by demographic groups (gender, age groups, province) for OPS and Hcp1 ELISA assays for both liberal ($\geq \mu + 2\sigma$) and conservative ($\geq \mu + 3\sigma$) cutoff values.

Table S6. Monte Carlo testing (999 permutations) of Moran's eigenvector maps (MEMs) describing positive spatial autocorrelation among the communes sampled. Values include the Moran's I statistic, z-score, and *p*-value ($\alpha < 0.05$). Significance is indicated with an asterisk.

Table S7. Monte Carlo testing (999 permutations) of spatial autocorrelation in continuous explanatory variables, including global tests and two-sided tests describing

positive (I+) and negative (I-) autocorrelation. Values include the Moran's I statistic, z-score, and *p*-value (α < 0.05). Significance is indicated with an asterisk.

Variable	Test	Moran's I	z-score	*p*-value
Average accumulated precipitation 2020-2022 (mm)	Global	0.92	32.06	0.001*
	I+	0.93	37.94	0.001*
	I-	-0.01	13.80	1.000
Cropland (%)	Global	0.65	22.26	0.001*
	I+	0.70	26.20	0.001*
	I-	-0.05	10.64	1.000
Distance to waterway (km)	Global	0.74	25.36	0.001*
	I+	0.77	29.34	0.001*
	I-	-0.03	12.49	1.000
Euclidean distance to provincial hospital (km)	Global	0.94	32.13	0.001*
	I+	0.95	37.41	0.001*
	I-	0.00	13.84	1.000
Nitrogen (cg/kg)	Global	0.36	12.19	0.001*
	I+	0.46	13.63	0.001*
	I-	-0.09	7.13	1.000
Organic Carbon (dg/kg)	Global	0.37	13.20	0.001*
	I+	0.48	14.13	0.001*
	I-	-0.10	6.36	1.000
Population density per km2	Global	0.47	16.09	0.001*
	I+	0.55	17.40	0.001*
	I-	-0.07	8.97	1.000
Soil moisture	Global	0.94	30.95	0.001*
	I+	0.95	37.93	0.001*
	I-	-0.01	14.17	1.000
Soil pH *10	Global	0.43	14.84	0.001*
	I+	0.52	17.09	0.001*
	I-	-0.09	7.22	1.000

Table S8. Forward selection of Moran's eigenvector maps (MEMs) identified nine significant MEMs each for null spatial models of liberal and conservative seropositivity. Both global Moran's I tests were significant (liberal: I = 0.181, $p = 0.0002$*; conservative: I = 0.192, $p = 0.0005$*). Significance is indicated with an asterisk.

Variable	R^2	Cumulative R^2	Adjusted cumulative R^2	*p*-value
Liberal Seropositivity				
MEM17	0.03	0.03	0.03	0.002*
MEM6	0.02	0.05	0.05	0.001*

MEM144	0.02	0.08	0.07	0.004*
MEM135	0.02	0.10	0.09	0.002*
MEM1	0.02	0.12	0.11	0.001*
MEM25	0.02	0.14	0.13	0.002*
MEM7	0.02	0.16	0.14	0.001*
MEM22	0.02	0.17	0.16	0.003*
MEM2	0.02	0.19	0.17	0.002*
Conservative Seropositivity				
MEM25	0.04	0.04	0.04	0.001*
MEM56	0.03	0.07	0.07	0.001*
MEM22	0.03	0.10	0.09	0.001*
MEM6	0.03	0.13	0.12	0.001*
MEM150	0.02	0.14	0.13	0.003*
MEM144	0.02	0.16	0.15	0.002*
MEM13	0.02	0.18	0.17	0.004*
MEM8	0.01	0.19	0.18	0.012*
MEM155	0.01	0.20	0.19	0.017*

Table S9. Final model validation using bootstrapping (n = 999 permutations). The likelihood ratio test χ^2 with degrees of freedom is provided as LR χ^2 and had a *p*-value of <0.0001 for both models. Apparent values were corrected by bootstrap optimism to adjust for modest overfitting identified by the calibration slope. Area under the curve (C-index) remains relatively high, indicating model stability despite a lower event per variable (EPV) value in the conservative model.

Metric	Liberal Seropositivity Model	Conservative Seropositivity Model
LR χ^2 (df)	102.8 (11)	96.5 (11)
Apparent C-index	0.79	0.85
Corrected C-index	0.76	0.82
Apparent R^2	0.30	0.37
Corrected R^2	0.23	0.30
Calibration slope	0.81	0.85
Calibration intercept	−0.20	−0.23
Corrected Brier score	0.14	0.08
