# Supplementary material for: Hospital‐Based Cross‐Sectional Study of Burkholderia pseudomallei Seroreactivity Among Febrile Patients in Northernmost Vietnam: Near‐Neighbor Bloodstream Isolates, Environmental Correlates, and Spatial Clustering
Source: Geohealth. 2026 May 30;10(6):e2025GH001606. doi: 10.1029/2025GH001606 (PMC13239667; doi:10.1029/2025GH001606)
Supplement: Supplementary file 2 — Table S1 [file GH2-10-e2025GH001606-s001.doc]

STROBE Statement

|  | Item No | Recommendation | Met? | Comments or statements |  |  |
| --- | --- | --- | --- | --- | --- | --- |
| **Title and abstract** | 1 | (*a*) Indicate the study’s design with a commonly used term in the title or the abstract | Yes | The study design, “a hospital-based cross-sectional study” is described in the title and abstract. L 35 |  |  |
|  |  | (*b*) Provide in the abstract an informative and balanced summary of what was done and what was found | Yes | This information is stated in the abstract. |  |  |
| Introduction | | |  |  |  |  |
| Background/rationale | 2 | Explain the scientific background and rationale for the investigation being reported | Yes | Rationale for studying this pathogen in general and in the region is stated in the introduction. “The epidemiology and geographic distribution of melioidosis in the northernmost provinces of Vietnam is poorly characterized”. L 138 -139 |  |  |
| Objectives | 3 | State specific objectives, including any prespecified hypotheses | Yes | The objectives are stated at the end of the introduction; “The objective of this study is two-fold; first, elucidate the distribution and environmental correlates of *B. pseudomallei* seroreactivity in the northernmost provinces of Vietnam; and second, to identify bacteria found in a subset of febrile hospital patients.” L 145 - 148 |  |  |
| Methods | | |  |  |  |  |
| Study design | 4 | Present key elements of study design early in the paper | Yes | The study design is described in the methods; first, that it is a hospital-based cross-sectional study (L 164 – 165) and second, that is it sourced from a previous surveillance effort in the region (L 163 – 164). |  |  |
| Setting | 5 | Describe the setting, locations, and relevant dates, including periods of recruitment, exposure, follow-up, and data collection | Yes | The study setting (hospital-based), location (six northernmost provinces), and recruitment time period (2020 – 2023) are explicitly stated in the methods section “Sample Collection and Management”. |  |  |
| Participants | 6 | (*a*) Give the eligibility criteria, and the sources and methods of selection of participants | Yes | The source of participants and the reasons for inclusion/exclusion in the study are described in the methods L 165 – 174. Detailed information on blood culturing inclusion was described on L 214 – 225. A flow diagram is also provided (Figure S7). |  |  |
| Variables | 7 | Clearly define all outcomes, exposures, predictors, potential confounders, and effect modifiers. Give diagnostic criteria, if applicable | Yes | The defined outcomes in this study (seropositivity) are described in the methods section “Seropositivity”. Potential confounders (such as age, gender, or geographic location) were assessed for significant differences between groups. These are described in Table 2 and Table S5. |  |  |
| Data sources/ measurement | 8* | For each variable of interest, give sources of data and details of methods of assessment (measurement). Describe comparability of assessment methods if there is more than one group | Yes | Data sources and their methods of pre-processing are explicitly described in the methods section “Commune-level environmental and geographic correlates” and in the Supporting Materials Table S3. Patients were spatially aggregated to their home commune (administrative level-3) for privacy. |  |  |
| Bias | 9 | Describe any efforts to address potential sources of bias | Yes | Bias was discussed in regard to healthcare accessibility (hospital-based convenience sampling; L 167 – 170) and the determined seropositivity cutoffs (lack of known positive/negative controls; L 273 – 291). |  |  |
| Study size | 10 | Explain how the study size was arrived at | Yes | Study size was not explicitly predetermined for this study but was derived from available sample from parent sera study; “Sample size was determined by the availability of specimens collected under the parent surveillance study; no *a priori* power calculation was performed”. L 174 – 176 |  |  |
| Quantitative variables | 11 | Explain how quantitative variables were handled in the analyses. If applicable, describe which groupings were chosen and why | Yes | Quantitative transformations: Continuous environmental variables were transformed (square root, log, logit) and scaled prior to regression. Detailed in methods section “Eigenvector-based spatial filtering and logistic regression” and Text S1.  Groupings: Continuous ELISA absorbance values were grouped into binary outcomes (seropositive/negative) using statistical thresholds (≥ µ + 2σ and ≥ µ + 3σ). This grouping was chosen to account for the lack of local negative controls and potential background noise in a febrile population. Age groups were also used in the study to produce stratified comparisons of seropositivity rates (L296 – 298). |  |  |
| Statistical methods | 12 | (*a*) Describe all statistical methods, including those used to control for confounding | Yes | Statistical tests were employed and described in the methods section; Chi-square with continuity correction (L 292 – 299) and spatially conditioned logistic regression in methods section “Eigenvector-based spatial filtering and logistic regression”. We addressed spatial confounding (autocorrelation) by using Moran’s eigenvector maps (MEMs) and spatial filtering. |  |  |
|  |  | (*b*) Describe any methods used to examine subgroups and interactions | Yes | Individuals were stratified by gender, age group, and home province. |  |  |
|  |  | (*c*) Explain how missing data were addressed | Yes | Missing data, specifically age and geographic location, led to exclusion from the study (Figure S7). Individuals with missing gender information were included in overall seropositivity modelling and mapping but excluded in demographic comparisons of seropositivity L 295 – 296. |  |  |
|  |  | (*d*) If applicable, describe analytical methods taking account of sampling strategy | N/A | N/A |  |  |
|  |  | (*e*) Describe any sensitivity analyses | N/A | No explicit sensitivity analyses were performed in this study. |  |  |
| Results | | |  |  |  |  |
| Participants | 13 | (a) Report numbers of individuals at each stage of study—eg numbers potentially eligible, examined for eligibility, confirmed eligible, included in the study, completing follow-up, and analysed | Yes | These details are described in the results section “Sampling demographics”. Detailed information on inclusion and exclusion is shown in a flow diagram (Figure S7). |  |  |
|  |  | (b) Give reasons for non-participation at each stage | Yes | Reasons for non-participation at each stage are described in “Sampling demographics” and a flow diagram (Figure S7). |  |  |
|  |  | (c) Consider use of a flow diagram | Yes | This was provided in Supporting materials (Figure S7) and noted in results L 408 – 409. |  |  |
| Descriptive data | 14 | (a) Give characteristics of study participants (eg demographic, clinical, social) and information on exposures and potential confounders | Yes | In Table 1, the characteristics of study population is described for the study area and within individual provinces (scale of collection i.e., provincial hospitals). The age median and IQR are described. The number and percentage of participants are also described within demographic groups (age and gender). |  |  |
|  |  | (b) Indicate number of participants with missing data for each variable of interest |  |  |  |  |
| Outcome data | 15 | Report numbers of outcome events or summary measures | Yes | Explicit reporting of outcome (seropositivity) following liberal and conservative definitions are detailed in the results section “Seropositivity”, L 475 – 483). Summary measures by demographic groups are detailed in Table 2, Table S5). Tables detail both seropositive and seronegative groups. |  |  |
| Main results | 16 | (*a*) Give unadjusted estimates and, if applicable, confounder-adjusted estimates and their precision (eg, 95% confidence interval). Make clear which confounders were adjusted for and why they were included |  | Estimates: Table 3 provides the raw coefficients (estimates) and SE. The text reports the converted Odds Ratios with 95% CIs (e.g., "precipitation (1.69, 1.21-2.39)"). Eigenvector modelling specifically adjusts for spatial autocorrelation (confounding) using MEMs (e.g., "MEM17", "MEM6"). Detailed information on MEM selection, model performance, and the successful correction for spatial autocorrelation in model residuals is discussed in Text S2. |  |  |
|  |  | (*b*) Report category boundaries when continuous variables were categorized |  | Serology: Explicit boundaries are stated for seropositivity classification: "surpassing 2.072 Au" (OPS) and "surpassing 0.362 Au" (Hcp1) L 480 – 481. Table 1 clearly defines the boundaries for the Age Groups (e.g., <1, 1-4, 5-9). |  |  |
|  |  | (*c*) If relevant, consider translating estimates of relative risk into absolute risk for a meaningful time period | N/A | N/A |  |  |
| Other analyses | 17 | Report other analyses done—eg analyses of subgroups and interactions, and sensitivity analyses | N/A | N/A |  |  |
| Discussion | | |  |  |  |  |
| Key results | 18 | Summarise key results with reference to study objectives |  | The discussion explicitly summarises seroprevalence, seasonality, ecological correlates, and isolation of related species, linking them directly to the study objectives. |  |  |
| Limitations | 19 | Discuss limitations of the study, taking into account sources of potential bias or imprecision. Discuss both direction and magnitude of any potential bias | Yes | Sources of potential bias (blood‑culture sensitivity, convenience sampling, spatial autocorrelation) and magnitude/direction of bias are acknowledged. |  |  |
| Interpretation | 20 | Give a cautious overall interpretation of results considering objectives, limitations, multiplicity of analyses, results from similar studies, and other relevant evidence | Mostly | Cautious language is used when appropriate “hypothesis generating”, “exploratory”. The inclusion of studies from other regions in Southeast Asia are used to support our interpretation. |  |  |
| Generalisability | 21 | Discuss the generalisability (external validity) of the study results | Yes | The study explicitly acknowledges that “he findings of this study cannot be extrapolated to the general population” and the reasoning for this L 672 – 676). |  |  |
| Other information | | |  |  |  |  |
| Funding | 22 | Give the source of funding and the role of the funders for the present study and, if applicable, for the original study on which the present article is based | Yes | Funding sources are stated in the “Acknowledgements” section. |  |  |
